# Methyl on the Bridge: 2‐Methyl Propellane as Precursor for 1,3‐Substituted 2‐Methylated Bicyclo[1.1.1]Pentanes

**DOI:** 10.1002/chem.202502396

**Published:** 2025-10-15

**Authors:** Sean R. Verschaeve, Mariana Gomes Manso, Tibo Van Eeckhoorn, Mark E. Light, Bruno Linclau

**Affiliations:** ^1^ Department of Organic and Macromolecular Chemistry Ghent University Campus Sterre S4, Krijgslaan 291 Ghent B‐9000 Belgium; ^2^ School of Chemistry and Chemical Engineering University of Southampton Highfield Southampton SO171BJ UK

**Keywords:** 2‐methyl propellane, bicyclo[1.1.1]pentane, lipophilicity, melting point, methyl

## Abstract

Bicyclo[1.1.1]pentanes (BCPs) have emerged as isosteric replacements for mono‐ and para‐substituted benzene rings in medicinal and materials applications, involving substitution at the BCP bridgehead (1,3‐positions). BCP functionalization at the 2‐position is much less straightforward and currently under intense investigation. Herein, we report a synthetic route to 2‐methyl BCPs allowing for functionalization at the 1,3‐positions, with the novel 2‐methyl[1.1.1]propellane as a key intermediate. Next to an optimization for the synthesis of 2‐Me‐propellane, this work contains an investigation of its reactivity leading to 1,2‐disubstituted and 1,2,3‐trisubstituted BCP derivatives. Compared to nonsubstituted propellane, the synthesis of 2‐methyl propellane was lower‐yielding, and its ring‐opening reactions proceeded in similar or lower yields, with radical‐based reactions generally giving the best results. A preliminary study of selected physicochemical properties was conducted to assess the impact of the introduction of a bridge‐methyl group, showing an expected increase of about 0.5 log*P* units and featuring lower melting points.

## Graphical abstract

The synthesis of 2‐methyl propellane and its conversion to various 1,2‐ and 1,2,3‐di‐ and trisubstituted bicyclopentanes is reported.

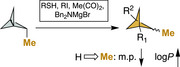

## 1 Introduction

**Figure 1:**
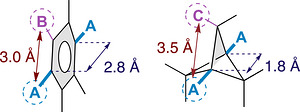
Bicyclopentanes as benzene isosteres, highlighting “*para”*‐ and “*ortho*”‐substitution.

In the drug optimization process, replacing a benzene ring in bioactive compounds by so‐called “3D‐bioisosteres” is a possible strategy to improve bioactivities and physicochemical properties.^[^ ^1^ ^]^ The bicyclo[1.1.1]pentane (BCP) ring has been extensively employed for this purpose.^[^ ^2^, ^3^, ^4^, ^5^, ^6^ ^]^ Applications involving monosubstituted BCP rings, typically as a phenyl biosiostere, and 1,3‐disubstituted BCP rings, as bioisostere of para‐substituted benzene rings, have been well‐described. For the latter, this is due to the identical spatial equivalence between the 1,3‐BCP and *para*‐benzene positions (Figure 1), although the distance is smaller for BCP derivatives. 1,3‐Disubstituted bicyclo[1.1.1]pentanes (BCPs) have also been used as bioisostere of disubstituted alkynes,^[^ ^7^ ^]^ and as alternative “spacer” for *para*‐substituted benzene rings and acetylenes in organic materials.^[^ ^2^, ^8^ ^]^ Another application of monosubstituted BCP rings is as *tert*‐butyl isostere.^[^ ^9^, ^10^ ^]^

There are far fewer examples containing bridge‐substituted BCP rings.^[^ ^11^, ^12^ ^]^ The possibility of 1,2‐disubstituted BCP rings as bioisosteric replacements of *ortho*‐substituted benzene rings was proposed in 2019.^[^ ^3^ ^]^ While the spatial orientation of the coplanar *ortho*‐substituents in benzene is clearly different from the 1,2‐substituents in BCP (Figure 1), their distance is similar (3.0 Å for two ortho‐methyl groups versus 3.5 Å for two 1,2‐BCP methyl groups).

**Scheme 1:**
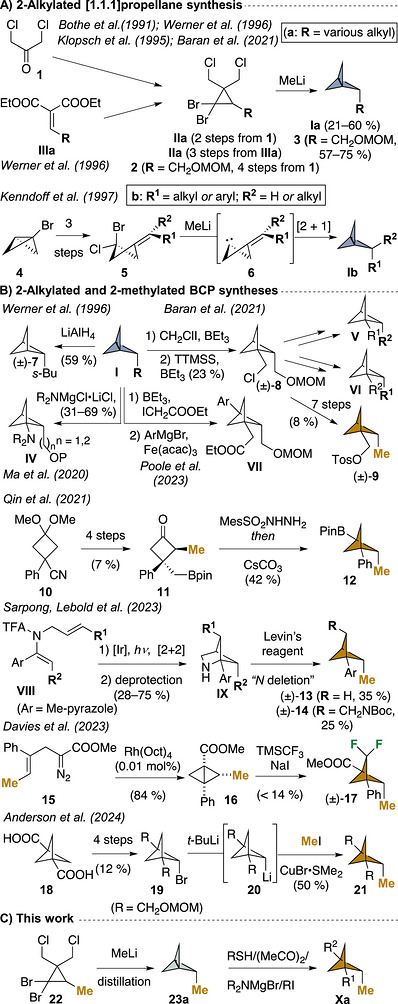
Precedence for the synthesis of 2‐substituted propellanes and bicyclopentanes.

**Scheme 2:**
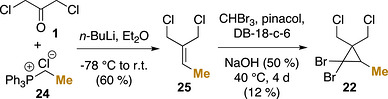
Synthesis of tetrahalide propellane precursor **22**.

Next to the methodology leading to BCPs with halogenation,^[^ ^13^, ^14^, ^15^, ^16^, ^17^, ^18^, ^19^ ^]^ or arylation ^[^ ^19^, ^20^, ^21^, ^22^, ^23^, ^24^, ^25^, ^26^ ^]^ at the 2‐position, several synthetic methodologies have been developed to obtain 2‐alkylated BCP rings.^[^ ^11^, ^12^, ^26^, ^27^ ^]^ The early examples are based on ring opening of 2‐alkylated [1.1.1]propellanes, for which various methodologies allowing for their large‐scale syntheses have been developed (Scheme 1A). In 1991, Bothe et al. reported a three‐step synthesis of monosubstituted [1.1.1]propellanes **Ia** based on a sequential bromine‐lithium exchange‐intramolecular chloride substitution sequence from tetrahalides **IIa** as cyclization precursors (Szeimies’ methodology).^[^ ^28^ ^]^ These were obtained via introduction of various alkyl groups (>4C) by Wittig olefination of 1,3‐dichloroacetone **1,** ^[^ ^29^ ^]^ followed by *gem*‐dibromocyclopropanation. Similarly, in 1995, Klopsch et al. reported a five‐step procedure to achieve a MOM‐protected 2‐hydroxymethyl[1.1.1]propellane **3**.^[^ ^30^ ^]^ Building on the work of Bothe et al., Werner et al. extended the alkyl series (>1C) and developed a three‐step synthetic protocol toward the substituted tetrahalide intermediate **IIa** starting from diethyl‐2‐alkylidene malonates **IIIa**.^[^ ^31^ ^]^ In 2021, the Baran group revised the five‐step Klopsch synthesis, improving the overall yield of the MOM‐protected 2‐hydroxymethyl[1.1.1]propellane **3** from 11 to 35%.^[^ ^32^ ^]^ Scheme 2, 7

In 1997, Kendoff et al. reported an alternative approach toward an extensive series of mono‐ or disubstituted [1.1.1]propellanes **Ib**. The six‐step synthetic protocol starting from 1‐bromobicyclo[1.1.0]butane **4** involved an in situ carbene formation **6** and consecutive intramolecular [2 + 1] cyclopropanation to yield the mono‐ or di‐alkylated and arylated [1.1.1]propellanes **Ib**.^[^ ^33^ ^]^

Despite these various established [1.1.1]propellane syntheses, only two of these had been converted to 2‐substituted BCPs at the time, both by reduction with LiAlH_4_, as illustrated by the synthesis of (±)‐**7** (scheme 1B).^[^ ^31^ ^]^ The other examples of ring opening of 2‐alkylated propellanes include a 2020 report from the Ma group with an impressive study for the synthesis of various 2‐substituted BCP derivatives **IV** through strain‐release amination^[^ ^34^ ^]^ of 2‐substituted propellane derivatives **I**.^[^ ^35^ ^]^ In the aforementioned study by Baran, radical‐mediated ring opening of methoxymethoxymethyl‐substituted [1.1.1]propellane **3** was reported using chloroiodomethane, with subsequent iodide reduction leading to (±)‐**8**, which was then converted to a series of 1,2‐disubstituted BCPs **V** and their “reversed bioisosteres” **VI**.^[^ ^32^ ^]^ The reduction of the MOM‐protected alcohol was also achieved, leading to the first methyl‐substituted BCP (±)‐**9**. The Poole group also used **3** for the synthesis of 1,2,3‐trisubstituted BCPs **VII**.^[^ ^23^ ^]^

The direct synthesis of 2‐methylated BCPs has been mostly reported as part of scope investigations during methodology developments. In 2021, the Qin group developed an original cyclization process starting from cyclobutane **10,** leading to 3‐Bpin‐substituted BCPs such as **12**.^[^ ^26^ ^]^ Electrophilic methylation allowed the synthesis of 2‐methylcyclobutanone derivative **11**, serving as the precursor for enantiopure 2‐MeBCP **12**, in low overall yield. A higher‐yielding (23%) nonstereoselective approach to (±)‐**12**, also starting from **10**, was later reported, with further extension of the methodology to 2‐Bpin‐substituted BCPs (not shown).^[^ ^21^ ^]^

In 2023, the groups of Sarpong and Lebold reported another conceptually different approach to 2‐alkylated BCPs via skeletal editing. Two examples of 2‐MeBCPs, **13** and **14**, were reported. They were obtained from enamides **VIII** by conversion to azabicyclo[2.1.1]hexanes **IX** via Ir‐catalyzed photochemical [2 + 2] cycloaddition and a deprotection step. The latter can be transformed into the corresponding 2‐alkylated BCPs via N‐atom deletion using the (expensive) Levin's reagent.^[^ ^22^ ^]^ Recently, the groups of Zhang and Lu and Tan reported similar N‐atom deletion approaches, although no methylation on the 2‐position was included.^[^ ^24^, ^25^ ^]^

In 2023, the Davies group reported an Rh‐catalyzed cyclization of the diazo compound **15**, leading to 2‐methyl [1.1.0]BCB **16** in excellent yield. Subsequent difluorocarbene addition afforded 2,2‐difluoro‐3‐methyl BCP derivative (±)‐**17** in low yield.

A final literature example of a 2‐methylBCP synthesis was reported by Anderson et al. in 2024 via anionic BCP bridge functionalization.^[^ ^20^ ^]^ This strategy is based on the radical BCP‐bridge C‐H bromination reported by the MacMillan group.^[^ ^19^ ^]^ Hence, 1,3‐BCP dicarboxylic acid **18** was transformed in four steps to the 2‐brominated BCP derivative **19**. Subsequent lithiation using *t*‐BuLi afforded the stabilized lithiated intermediate **20**, which could be methylated to afford **21** in 50% yield.

The methyl group is a very common substituent in pharmaceuticals, and its introduction in bioactive compounds typically results in a plethora of effects that are relevant in the drug discovery process.^[^ ^36^, ^37^, ^38^, ^39^ ^]^ The term “magic methyl effect” has been coined to describe the sometimes dramatic improvements that can be seen upon its introduction. Hence, comparing bioactive compounds containing BCPs with their methylated BCP analogues will be of great interest. For such a purpose, the availability of synthetic methodology that introduces diversity on a methylated BCP precursor would be advantageous. Herein we report our efforts toward developing a versatile methodology for the synthesis of bridge‐methylated BCPs **Xa** (Scheme 1C). The novel 2‐methyl[1.1.1]propellane **23a** was deemed an ideal intermediate for this purpose, as it allows mono‐ and difunctionalization of its central 1,3‐bond. Next to the synthesis of **23a**, an investigation of its reactivity compared to that of nonsubstituted [1.1.1]propellane **23b** was undertaken. We also report our preliminary results regarding the impact of BCP 2‐methylation on physical properties such as melting point and lipophilicity.

## 2 Results and Discussion

### 2.1 Synthesis of 2‐methyl[1.1.1]Propellane 23a

The synthesis of **23a** was envisioned using the conventional lithiation procedure (Scheme 1C),^[^ ^7^, ^28^ ^]^ identifying **22** as the key intermediate. This was first synthesized by a Wittig reaction between 1,3‐dichloroacetone **1** and ethyl triphenyl phosphonium chloride **24**, leading to **25** (Scheme 2). It was found that the use of the chloride salt^[^ ^40^ ^]^ gave a much better yield compared to the reported^[^ ^41^ ^]^ use of the corresponding bromide salt, as it avoided the formation of allylic bromide side products (detected by MS‐analysis; see Supporting Information section 3.3). Despite much experimentation, however, the yield of the dibromocyclopropanation of **25** using bromoform could not be optimized, with the conditions reported by the Baran group in his synthesis of the MOMOCH_2_‐substituted propellane **2** (55% yield, cf Scheme 1A)^[^ ^32^ ^]^ only leading to a 12% yield.

An alternative route starting from the commercially available diethyl 2‐ethylidenemalonate **26** was more successful (Scheme 2). From **26**, the corresponding dibromocyclopropane **27** was obtained in 69% yield using Baird's procedure,^[^ ^42^, ^43^ ^]^ with careful workup to avoid ester saponification. This reaction could be optimized on 200 g scale, employing high‐vacuum distillation to isolate **27** in 72% yield. Subsequent ester reduction using a combination of LiAlH_4_ and AlCl_3_, which results in the formation of AlH_3_, afforded diol **28** in 49% yield (see Supporting Information section 3.4). Similar yields were reported for higher alkylated cyclopropane derivatives.^[^ ^31^ ^]^

**Scheme 3:**
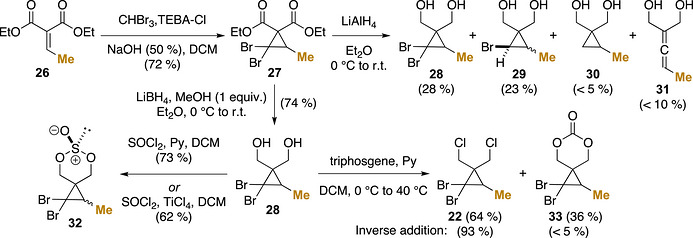
Optimized synthesis of the tetrahalide propellane precursor **22**.

The low yield of the ester reduction was initially attributed to significant product loss during aluminum salt removal in the workup. Using LiAlH_4_ as a reductant led to swift consumption of starting material at 0 °C, but the monobrominated diastereomers **29** (Scheme 3) were isolated as major side products, with the major isomer assigned as the *cis*‐isomer (70:30) based on coupling constant analysis (see Supporting Information section 3.3.3). Two other side products were characterized as the fully debrominated **30** and the allene **31**. Dibromocyclopropane reduction has also been reported by Baird et al. on the nonalkylated dibromocyclopropane substrate,^[^ ^44^ ^]^ which they showed could be avoided by using the milder alane (AlH_3_). In our hands, repeated efforts of applying alane for the selective reduction remained unsuccessful, with debromination still observed. In fact, TLC analysis revealed that debromination occurred before complete consumption of the starting material.

To prevent debromination, the use of milder metal borohydrides was explored. The use of LiBH_4_ in Et_2_O at room temperature resulted in incomplete conversion of the starting material, with the formation of debrominated side products **29** upon longer reaction times or applying reflux conditions. To our delight, the addition of methanol in stoichiometric amounts, known to increase the reactivity of LiBH_4_,^[^ ^45^ ^]^ resulted in fast and complete conversion of the starting material with limited formation (< 15%) of side products, which were difficult to separate. Furthermore, the workup is more convenient compared to reactions with aluminum hydrides. On a 20‐gram scale, purification with a single flash chromatographic separation resulted in 74% of the pure desired diol **28**, next to a small amount of a mixture of **28** and **29**. Efforts to completely prevent debromination using the significantly less expensive NaBH_4_ resulted in incomplete conversion of the starting material and long reaction times, while still observing debromination.

Bis‐deoxychlorination of diol **28** using the conventional Appel conditions^[^ ^31^ ^]^ surprisingly led to a complex mixture from which the desired tetrahalide **22** could not be isolated (see Supporting Information section 3.3). Given that these conditions lead to the formation of two equivalents of triphenyl phosphine oxide and require the use of restricted CCl_4_ as a solvent, it was decided to explore other chlorination methodologies.

The use of Vilsmeier‐Haack (POCl_3_) procedures^[^ ^46^ ^]^ also led to complex mixtures (see Supporting Information section 3.3), and Darzens chlorination conditions (thionyl chloride/pyridine or thionyl chloride/TiCl_4_^[^ ^47^ ^]^) led to the formation of a mixture of the corresponding diastereomeric bicyclic spiro sulfite esters **32**. A successful reaction was achieved using a method reported by Kartika et al., employing triphosgene‐pyridine.^[^ ^48^, ^49^ ^]^ yielding the desired compound **22**. However, the corresponding bicyclic spiro carbonate **33** was also isolated. The ratio **22**/**33** varied from run to run, but the carbonate was usually isolated as the minor product, in accordance with the results obtained by Kartika. We hypothesized that the formation of the carbonate by intramolecular ring closure competes with the intermolecular S_N_2 displacement of the activated alcohol by a chloride anion. Presumably, the all *cis*‐configuration of the bromide and methyl groups enhances steric hindrance for S_N_2 displacement. To facilitate intermolecular chloride attack, more concentrated reaction conditions were applied but with limited effect. However, by applying a reverse addition, in which the —barely soluble— diol **28** was added to a mixture of triphosgene and pyridine, proved to be an efficient solution. This procedure ensures a continuous excess of chlorination reagent, efficiently outcompeting intramolecular ring closure and significantly improving the yield of the desired tetrahalide from **22** to 93%.

**Scheme 4:**
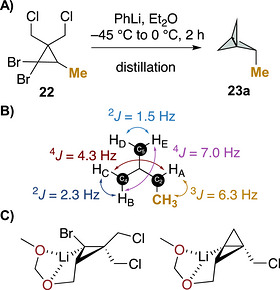
Synthesis of 2‐Me propellane **23a**.

With an optimized synthesis of **22** in hand, the synthesis of 2‐Me‐propellane **23a** was performed using methods reported for the nonsubstituted propellane (Scheme 4A).^[^ ^50^, ^51^ ^]^ Hence, consecutive bromine‐lithium exchange–S_N_2‐chloride displacement reactions resulted in 2‐Me‐propellane **23a** formation. Purification was achieved by distillation to ensure the safe collection of the unstable product, which was calculated to have a slightly enhanced ring strain compared to nonsubstituted propellane,^[^ ^52^ ^]^ as an ethereal solution. For this process, we obtained **23a** in concentrations of 0.12 M to 0.24 M in Et_2_O in varying yields (14–39%, NMR yield). Full NMR characterization of **23a** was achieved (Scheme 4B, Supporting Information section 2), which showed the usual vicinal (^2^ *J*_HH_) and long‐range (^4^ *J*_HH_) coupling constants involving the ring‐hydrogens.

These obtained yields are much lower compared to those for the nonmethylated propellane **23b** (up to 91%), obtained using the same procedure and setup. Nevertheless, these yields are in line with the closely related 2‐substituted propellanes reported by Werner et al. for the 2‐ethyl[1.1.1]propellane (16%) and 2‐*n‐*propyl[1.1.1]propellane (45%).^[^ ^31^ ^]^ We suggest this is due to the methyl group causing steric hindrance in the ring‐closing step that takes place at the same face of the three‐membered ring. In contrast, the synthesis of the MOM‐protected hydroxy 2‐substituted propellane **3** by the Baran group proceeded in 75%,^[^ ^32^ ^]^ improving the earlier reported yield of 57%.^[^ ^30^ ^]^ We presume this is due to the possibility of MOM‐mediated chelation, as suggested by Anderson et al. for similar lithiations on the 2‐position (Scheme 4C), in combination with a lower volatility.^[^ ^20^ ^]^

### 2.2 Reactivity of 2‐methyl [1.1.1]Propellane 23a

**Scheme 5:**
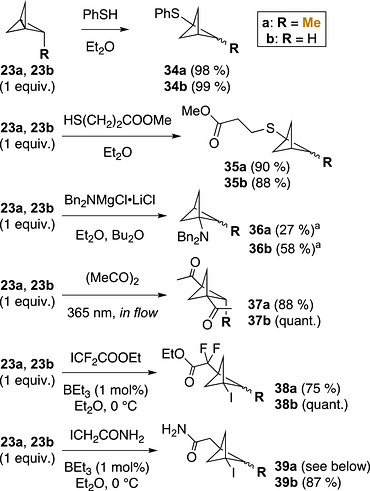
Functionalization of 2‐Me[1.1.1]propellane **23a** and propellane **23b**. Yields are isolated yields. See supporting information for the reactions with propellane. ^a^ Yield including propellane formation and amination.

With **23a** in hand, its reactivity was investigated under a number of known propellane ring‐opening reactions (Scheme 5). In all cases, **23a** was used as the limiting reactant, and the corresponding yield of the reaction with the nonsubstituted propellane **23b** is provided for comparison.

It was found that **23a** reacted swiftly with thiophenol^[^ ^28^, ^53^ ^]^ to give thioether **34a** in near‐quantitative yields (98%). Similar results were obtained for the reaction with methyl 3‐mercaptopropionate that led to **35a** in 90% yield. These yields are similar to the yields of **34b** and **35b**, which are obtained from the nonbridge‐functionalized propellane **23b**. The procedure for Baran's strain release amination^[^ ^34^ ^]^ was also applied to 2‐Me‐propellane **23a**, although the solvent was changed to Et_2_O since the starting material (**22**) was not soluble in Bu_2_O at low temperatures. The dialkylaminobicyclo[1.1.1]pentane **36a** was obtained in only 27% yield, a lower yield than the reported yield obtained for the nonbridge‐substituted BCP **36b** (58%, lit^[^ ^34^ ^]^ 54%). Photochemical ring opening of propellane **23a** with 2,3‐butadione *in flow*, as reported by Ripenko et al. using 365 nm irradiation at room temperature,^[^ ^54^ ^]^ afforded 1,3‐diacyl‐2‐methyl BCP **37a** in 88% yield. However, the nonmethylated derivative **37b** can be obtained in quantitative yield, without the need for a purification step, whereas side product formation occurs with 2‐Me‐propellane **23a**, resulting in the need for flash column chromatography. Lowering the temperature to −20 °C, a lower 62% yield for **37a** was obtained, still with the formation of a number of unidentified side products. At −20 °C, a still quantitative yield for **37b** was obtained.

**Scheme 6:**
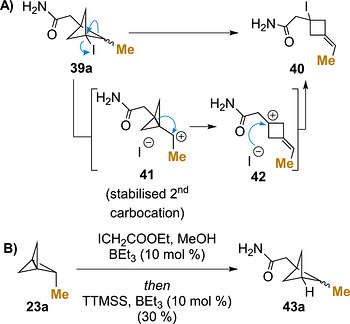
A) Proposed rearrangement for the formation of **40**; B) radical addition with in‐situ iodide reduction.

Triethylborane‐initiated atom‐transfer radical addition with ethyl iododifluoroacetate as developed by Anderson et al.^[^ ^55^ ^]^ led to iodide **38a** in good yield, compared to a quantitative yield when using nonsubstituted propellane. The corresponding radical addition of (nonfluorinated) iodoacetamide with propellane **23b** gave **39b** in excellent yield after purification by column chromatography. The same reaction with 2‐Me propellane **23a** resulted in the formation of **39a** (Supporting Information section 5.13.1 for crude ^1^H NMR‐spectrum), but purification by flash column chromatography led to complete degradation (^1^H NMR and LC‐MS analysis). Instead, a ring‐opened iodo cyclobutane **40** was isolated in 30% yield (Scheme 5, 6), whose formation can be rationalized by ionic cleavage of the carbon‐iodine bond resulting in a bicyclobutane intermediate **41**, followed by rearrangement to the more stable **42**. Poole et al. reported occasional rapid decomposition of similar BCP iodides (with a 2‐MOMO‐methyl group) following column chromatography.^[^ ^23^ ^]^

When the reaction mixture was immediately treated with TTMSS/BEt_3_ at room temperature (Scheme 6B), a complex reaction mixture was obtained, from which the reduction product **43a** was isolated via preparative RP‐HPLC in 30% yield, next to unidentified side products. Bench‐stable bridge‐substituted BCP‐iodides that do not have alkylation at the 2‐position have been reported to undergo such cationic ring‐opening reactions when initiated with AgSbF_6_.^[^ ^56^ ^]^ With bridge‐alkylation, exposure to silica gel suffices to initiate the ring‐opening, given the formation of a more stabilized secondary cation intermediate **40**. This process was not observed in the reaction with ethyl iododifluoroacetate leading to iodide **38a**, which is explained by the fluorine electron‐withdrawing effect destabilizing the formation of cationic species. Hence, reactions of the nonsubstituted [1.1.1]propellane **23b** consistently proceed in higher yields compared to **23a**.

**Scheme 7:**
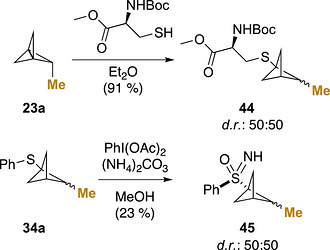
Reactions leading to diastereomeric products.

Unlike for [1.1.1]propellane **23b,** ring opening of 2‐methyl[1.1.1]propellane **23a** with chiral reagents will result in diastereomers. This was investigated as shown in Scheme 7. Reaction of **23a** with protected l‐cysteine led to a 1:1 mixture of inseparable diastereoisomers **44** in 91% yield. Equally, oxidation of sulfide **34a** using the method of Li^[^ ^57^ ^]^ led to the corresponding sulfoximine diastereomers **45** without stereoinduction. The Bräse group had reported a 55% yield for the oxidation of the corresponding nonmethylated phenyl BCP sulfide **34b**.^[^ ^58^ ^]^ Neither diastereomeric mixture **44** nor **45** could be separated using normal‐phase silica gel chromatography.

### 2.3 Investigation of Physical Properties

**Figure 2:**
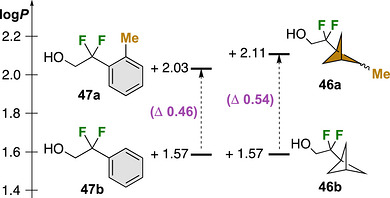
Comparison of measured octanol‐water partition coefficients between phenyl, tolyl, BCP, and 2‐MeBCP.

The effect of the added bridge‐methyl group on physicochemical properties was investigated. The lipophilicity of BCP **46b** and its aromatic equivalent **47b**, measured by a ^19^F NMR‐based protocol developed in our group,^[^ ^59^ ^]^ was shown to be identical (Figure 2), while addition of a methyl group (**46a**) led to a significant increase of 0.54 log*P* units. This increase in lipophilicity is similar to the effect for the addition of a methyl group in *ortho*‐position (**47a**, 0.46 log*P* unit) and what is reported in the literature,^[^ ^37^ ^]^ including for close linear 2,2‐difluoroalkanol analogues (Δlog*P* between 2,2‐difluorobutanol and 2,2‐difluoropentanol is 0.58 log*P* units).^[^ ^60^ ^]^ This result is in line with the increase predicted by conventional clog*P* methodologies (see Supporting Information section 1.7).

**Figure 3:**
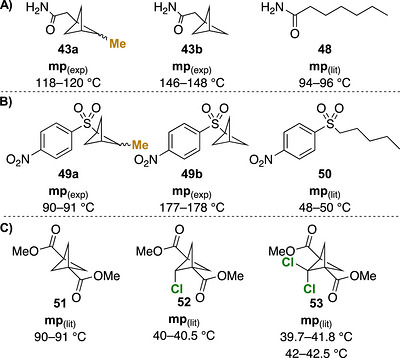
Melting point comparison of bridge‐substituted BCPs with their nonbridge‐substituted analogues.

1,3‐Disubstituted bicyclopentanes are often solid compounds. In contrast, most of the 2‐Me‐BCPs were obtained as liquids, probably due to the absence of symmetry leading to less favorable crystal packing. For example, whereas **37b** and **38b** were isolated as crystalline solids, their corresponding 2‐Me analogues were liquids at room temperature. However, 2‐methylated BCP derivatives **43a** and **49a** were found to be solids (Figure 3A/B), with melting points much lower than that of the nonmethylated equivalents, especially for **49a**/**49b** (ΔT = 87 °C). The melting point of the corresponding *n‐*alkyl sulfone **50** is lower than that of the BCPs **49a** and **49b**, as is the melting point of **48** compared to that of **43a**/**43b**. This is in agreement with the typically lower melting points of open‐chain alkanes compared to cyclic structures. Such melting point reduction upon bridge functionalization can also be found upon chlorination (Figure 3C). Whereas dimethyl bicyclopentane dicarboxylate **51** has a melting point of 90–91 °C,^[^ ^61^, ^62^ ^]^ this is much reduced for the monochlorinated derivative **52**.^[^ ^13^ ^]^ The melting point of the bridge‐dichlorinated **53** is similar to that of **52**. ^41] [^ ^63^ ^]^

**Scheme 8:**
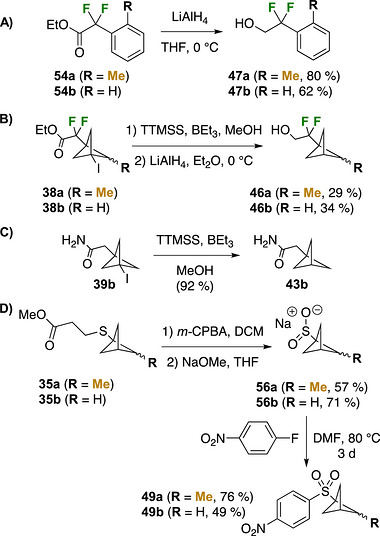
Syntheses of substrates **43a**‐**49b**.

The syntheses of the novel substrates shown in Figures 2 and 3 are shown in Scheme 8. The aromatic log*P* substrates **47a** and **47b** were obtained by reduction of the corresponding ethyl esters **54a** and **54b**, respectively (Scheme 8A), which were either synthesized according to a reported method (**54a**),^[^ ^64^ ^]^ or were commercially available (**54b**). Substrates **46a** and **46b** were obtained by radical‐mediated iodine reduction^[^ ^55^ ^]^ of **38a** and **38b**, respectively, in near‐quantitative yields, followed by ester reduction (Scheme 8B). Acetamide **43b** was obtained, similarly to **43a**, by reduction of iodide **39b**, following the procedure by Anderson et al.^[^ ^55^ ^]^ (Scheme 8C) Oxidation of sulfide **35a** to the corresponding sulfone **55a** using *m*‐CPBA, followed by a retro‐Michael reaction^[^ ^65^ ^]^ led to the sodium sulfinate **56a** in a combined yield of 57%. The corresponding process starting from **35b** had been originally reported by the Bräse group in 2020, in an overall yield of 82%,^[^ ^66^ ^]^ which we successfully reproduced (81% yield). S_N_Ar reaction of **56a** with 1‐fluoro‐4‐nitrobenzene afforded a crystalline racemic mixture **49a,** in 76% yield. The corresponding non‐functionalized BCP **49b** was obtained from **35b** in 35% yield over three steps.

## 3 Conclusions

In conclusion, we have developed a practical 4‐step synthesis for 2‐methyl[1.1.1]propellane and explored its value as a precursor for 2‐methylated BCPs. Careful optimization of an ester reduction step proved difficult due to an unavoidable debromination side reaction but could be achieved using LiBH_4_/MeOH as an active reductant. A remaining challenge is the low‐yielding propellane formation, which may be due to inherent steric hindrance of the methyl group and which is also observed with larger alkyl groups. Investigation of the reactivity of 2‐methyl [1.1.1]propellane with various ring‐opening reaction conditions showed that radical‐mediated ring‐opening reactions generally proceeded with similar yields compared to non‐functionalized [1.1.1]propellane, whereas other processes proceeded with lower yields. Noteworthy is the observed instability of 2‐methyl BCP iodides to acidic conditions, resulting in ring‐opening and rearrangement to the corresponding cyclobutane derivative.

We did not observe any stereoselectivity when 2‐methyl[1.1.1]propellane was reacted with a chiral thiol or in the sulfoximine formation of a phenyl‐2‐MeBCP sulfide. A preliminary investigation of the impact of a bridge‐methyl on physicochemical properties showed that the introduction of a methyl group on the bridge results in an expected increase in lipophilicity of 0.5 log*P* units and a decrease in melting point compared to their non‐functionalized BCP analogues.

Despite the moderate yield of the formation of 2‐methyl propellane, the overall synthetic efficiency of 2‐methyl BCP formation (5 steps from a cheap starting material) compares well with other reported methodologies (as these often also include a low‐yielding step). Hence, this methodology will be of interest for the synthesis of bridge‐methyl‐substituted bicyclopentanes.

## 4 Experimental Section

### 4.1 General Synthesis Information

Chemical reagents were obtained from commercial sources and used without further purification, unless stated otherwise. Anhydrous solvents were distilled immediately prior to use, except for anhydrous DMF, which was purchased in sealed containers from commercial sources. Et_2_O and THF were distilled from Na/benzophenone immediately prior to use. DCM and Et_3_N were dried over CaH_2_. All glassware was flame‐dried under vacuum and cooled under N_2_ prior to use. Water‐or air‐sensitive reactions were performed under an inert atmosphere, using dry solvents. Reactions were monitored by TLC (Merck Kieselgel 60 F254, aluminum sheet). Column chromatography was performed on silica gel (60 Å, particle size 35–70 µm). All reported solvent mixtures are volume measures. Preparative RP‐HPLC was carried out using a Phenomenex Luna Axia C_18_ column (250×22 mm, particle size 5 µm at 17.5 mL min^−1^, 35 °C). ^1^H, ^19^F, ^13^C NMR spectra were recorded at room temperature on a BRUKER AV400/500 spectrometer. ^1^H and ^13^C chemical shifts (δ) are quoted in ppm relative to residual solvent peaks as appropriate. ^19^F spectra were externally referenced to CFCl_3_. The coupling constants (*J*) are given in Hertz (Hz). The proton NMR signals were designated as follows: s (singlet), d (doublet), t (triplet), q (quartet), quin (quintet), sxt (sextet), spt (septet), m (multiplet), br (broad), app (apparent), or a combination of the above. IR spectra were recorded as neat films on a Nicolet 380 FT‐IR. Absorption peaks are given in cm^−1^ and the intensities were designated as follows: w (weak), m (medium), s (strong), br (broad). Melting points were recorded on a Reichert melting point apparatus, equipped with a Reichert microscope. Low‐resolution ES mass spectra were recorded on a WATERS ZMD single quadrupole system. High‐resolution mass spectra were recorded on a Bruker Apex III FT‐ICR‐MS and Agilent 6220a series TOF (LC‐HRMS) or Thermo MAT 900 XP double‐focusing sector field GC‐HRMS.

NOTE: For the 2‐MeBCPs, atom numbering is as follows: C_1_ is the higher‐priority bridgehead carbon, and C_2_ is the higher‐priority bridge carbon. The bridge carbon located at the *endo‐*position is C_4_.

### 4.2 Synthesis of Novel Compounds

#### 4.2.1 Synthesis of 1,1‐dibromo‐2,2‐bis(chloromethyl)‐3‐methylcyclopropane **22**

Under an anhydrous atmosphere, triphosgene (10.00 g, 34.3 mmol, 2.0 equiv.) was dissolved in anhydrous DCM (50 mL). The solution was cooled to 0 °C. Anhydrous pyridine (5.5 mL, 68.8 mmol, 4.0 equiv.) was added dropwise over 20 minutes. The mixture was stirred for 15 minutes at 0 °C, before the dropwise addition of a solution of **28** (4.70 g, 17.2 mmol, 1.0 equiv.) in anhydrous DCM (75 mL). The reaction was stirred for 10 minutes at 0 °C and then warmed to reflux. After 4 hours at reflux, the reaction was cooled to r.t. and diluted with DCM (150 mL). Diluted HCl (1 M, 175 mL) was carefully added. The phases were separated, and the organic layer was washed with NaHCO_3_ (sat. aq. sol., 150 mL). The organic layer was dried with MgSO_4_, filtered over celite, and concentrated. The crude mixture was filtered over silica gel (*n*‐pentane 100%) and concentrated to afford **22** (4.98 g, 16.0 mmol, 93%) as white crystals. ***R*_f_** = 0.41 (*n*‐pentane 100%); **m.p**. (not recrystallized): 38–40 °C; **^1^H NMR** (500 MHz, benzene‐*d*_6_): δ 3.78 (d, *J *= 11.8 Hz, 1H; CH_2_), 3.73 (d (roofed), *J *= 12.1 Hz, 1H; CH_2_), 3.39 (dd, *J *= 11.8, 1.1 Hz, 1H; CH_2_), 3.37 (dd, *J *= 12.0, 1.1 Hz, 1H; CH_2_), 0.96 (q (roofed), *J *= 6.5 HZ, 1H; CH), 0.71 (d (roofed), *J *= 6.6 Hz, 3H; CH_3_) ppm; **^13^C{^1^H} NMR** (101 MHz, benzene‐*d*_6_): δ 50.0 (CH_2_), 44.4 (CH_2_), 41.5 (CBr_2_), 36.8 (C), 35.9 (CH), 11.8 (CH_3_) ppm; **IR** (neat): 2932 (m), 758 (s), 693 (s), 588 (s) cm^−1^; **HRMS** (EI): *m/z* calcd for C_6_H_8_^79^Br_2_^37^Cl_2_: 307.8364 [*M*]^+.^, found 307.8352 (4.00 ppm error).

#### 4.2.2 Synthesis of 2‐methyl[1.1.1]Propellane **23a**

Tetrahalide **22** (5.71 g, 18.4 mmol, 1.0 equiv.) was ground into a grinder and placed in a high vacuum for 1 hour. After the flask was placed under an argon atmosphere, and anhydrous Et_2_O (30 mL) was added. The mixture was stirred at r.t. until all solids were dissolved and then cooled to −45 °C. Phenyllithium (19.30 mL, 36.74 mmol, 1.9 M in Bu_2_O, 2.0 equiv.) was added dropwise using a syringe pump over 15 minutes. When the addition was complete, the mixture was stirred for 5 minutes at −45 °C and then warmed to 0 °C. After stirring for 2 hours, a distillation apparatus flushed with argon was attached, and distillation was started using reduced pressure. 2‐Methyl [1.1.1]propellane **23a** was collected as a solution in Et_2_O (30 mL). Quantification of the amount of **23a** was achieved by ^1^H NMR analysis, using 50 µL of DCE as an internal standard, 200 µL of the collected solution, and CDCl_3_. The final concentration, calculated based on the ratio DCE:2‐methy[1.1.1]propellane (using the H^A^ signal), was 0.238 M corresponding to 573 mg, 7.15 mmol, 39%. Repetition of this process afforded 2‐methyl [1.1.1]propellane **23a** in concentrations of 0.12 M to 0.24 M in Et_2_O in varying yields (14–39%). Due to the instability of the compound, limited experimental data were obtained. **^1^H NMR** (500 MHz, CDCl_3_): δ 2.65 (dd, *J *= 7.0, 2.0 Hz, 1H; H^B^), 2.59 (qd, *J *= 6.3, 4.5 Hz; H^A^), 2.08 (dd, *J *= 4.6, 2.3 Hz; H^C^), 1.68 (dd, *J *= 7.0, 1.5 Hz; H^E^), 1.66 (d, *J *= 1.5 Hz; H^D^), 0.99 (d, *J *= 6.3 Hz, 3H; CH_3_) ppm; **^13^C{^1^H} NMR** (101 MHz, CDCl_3_): δ 84.2 (C_2_), 71.5 (C_4_), 69.0 (C_5_), 9.4 (CH_3_) ppm.

#### 4.2.3 Synthesis of 1‐chloro‐2‐(chloromethyl)but‐2‐ene **25**

To a flame‐dried flask under inert atmosphere containing a solution of ethyl triphenyl phosphonium chloride **24** (16.5 g, 55.1 mmol, 1.0 equiv.) in anhydrous Et_2_O (300 mL) was added dropwise *n*‐BuLi (2.5 M in hexanes, 20 mL, 55.1 mmol, 1.0 equiv.) at room temperature. After stirring for 1 hour, the reaction mixture was cooled to −78 °C, and a solution of 1,3‐dichloroacetone **1** (7.00 g, 55.1 mmol, 1.0 equiv.) in anhydrous Et_2_O (200 mL) was added dropwise. The reaction mixture was slowly warmed to r.t. and stirred overnight. The solution was filtered through a pad of Celite eluting with additional Et_2_O (400 mL) and concentrated. Purification through flash chromatography (SiO_2_, 100% pentane) afforded **25** (4.61 g, 33.1 mmol, 60%) as a colorless liquid. ***R*_f _** = 0.52 (*n*‐pentane 100%); **^1^H NMR** (400 MHz, CDCl_3_) δ 5.87 (q, *J* = 7.0 Hz, 1H; CH), 4.26 (s, 2H; CH_2_Cl), 4.20 (s, 2H; CH_2_Cl), 1.79 (d, *J *= 7.0 Hz, 3H; CH_3_) ppm; **^13^C{^1^H} NMR** (101 MHz, CDCl_3_) δ 133.3 (C), 131.0 (CH), 47.5 (CH_2_), 38.8 (CH_2_), 13.6 (CH_3_) ppm; **IR** (neat): 2959 (w), 1665 (m), 794 (s), 725 (s), 669 (s) cm^−1^; **MS** (CI) *m/z* (%) 138, 140 and 142 ([C_5_H_8_^35^Cl_2_, C_5_H_8_^35^Cl^37^Cl and C_5_H_8_^37^Cl_2_]^+.^, 28:9:6:1 ratio), 67 ([*M*‐Cl_2_]^+.^, 100); **HRMS** (EI): *m/z* calcd for C_5_H_8_^35^Cl_2_ 137.9998 [*M*]^+.^, found 138.0024 (‐2.7 ppm error).

#### 4.2.4 Synthesis of Diethyl 2,2‐dibromo‐3‐methylcyclopropane‐1,1‐dicarboxylate **27**

A three‐neck 4 L flask, equipped with a mechanical overhead stirrer and a pressure‐equalizing funnel, was charged with diethyl ethylidenemalonate **26** (150.0 g, 0.81 mol, 1.0 equiv.), bromoform (105.7 mL, 1.21 mol, 1.5 equiv.), and benzyl triethylammonium chloride (18.3 g, 80.6 mmol, 0.1 equiv.) in DCM (1.2 L). The mixture was ice‐cooled to 0 °C, and under fast stirring (400 rpm), a 50 w/w% NaOH solution (600 mL) was added dropwise via the addition funnel. After the addition of the 50 w/w% NaOH solution, the reaction was allowed to react at 0 °C for 10 minutes. The cooling bath was removed, and the reaction was allowed to stir at r.t. After complete conversion of the starting material (2.5 hours, TLC monitoring), water was added carefully (600 mL) (CAUTION: dilution of the caustic solution is exothermic!), and the phases were carefully separated. The aqueous phase was extracted with DCM (3 × 500 mL). The combined organic phases were dried with Na_2_SO_4_, filtered over a plug of silica, and concentrated. Purification through fractional high‐vacuum distillation (heating mantle, 10 cm Vigreux column) and collecting the fraction between 85–88 °C/0.085 Torr afforded **27** as a colorless liquid (207.7 g, 580.1 mmol, 72%, 99.3% GC‐purity). **B.p**. (0.05 Torr): 85–88 °C; **^1^H NMR** (500 MHz, CDCl_3_) δ 4.29 (q, *J *= 7.2 Hz, 2H; CH_2_), 4.28 (q, *J *= 7.2 Hz, 2H; CH_2_), 2.56 (q, *J *= 6.6 Hz, 1H; CH), 1.34 (d, *J *= 6.6 Hz, 3H; CHCH_3_), 1.33 (t, *J *= 7.2 Hz, 3H; CH_3_), 1.31 (t, *J *= 7.2 Hz, 3H; CH_3_) ppm; **^13^C{^1^H} NMR** (101 MHz, CDCl_3_) δ 165.2 (CO), 163.3 (CO), 62.9 (CH_2_), 62.0 (CH_2_), 44.6 (COCCO), 35.3 (CH), 31.6 (CBr_2_), 14.1 (CH_2_ CH_3_), 14.1 (CH_2_ CH_3_), 13.2 (CHCH_3_) ppm; **MS** (EI, 70 eV) *m/z* (%): 358, 360 and 362 (1) [*M*]^+.^, 249 and 251 (34), 141 (100) [*M* – Br_2 –_ Et_2_]^+.^ Data consistent with data in literature.^[^ ^43^ ^]^

#### 4.2.5 Synthesis of (2,2‐dibromo‐3‐methylcyclopropane‐1‐diyl)Dimethanol **28**

To a flame‐dried flask under argon was added **27** (8.33 g, 23.3 mmol, 1.0 equiv.) in anhydrous Et_2_O (50 mL). The solution was cooled to 0 °C, and a previously prepared solution of LiBH_4_ (2.03 g, 93.2 mmol, 4 equiv.) in anhydrous Et_2_O (50 mL) was added dropwise. Anhydrous methanol (0.94 mL, 23.2 mmol, 1.0 equiv.) was added dropwise, and the reaction mixture was allowed to warm to r.t. and stirred for 2 hours (TLC monitoring). The reaction mixture was cooled once again to 0 °C and quenched with NH_4_Cl (sat. aq. sol., 50 mL). The phases were separated, and the aqueous phase was extracted with EtOAc (3 × 60 mL). The combined organic phases were washed with brine, dried with MgSO_4_, filtered, and concentrated. Purification through flash chromatography (SiO_2_, cyclohexane/EtOAc 55:45) afforded **28** (4.63 g, 17 mmol, 74%). ***R*_f_** = 0.22 (DCM/MeOH 97:3); **m.p**. (not recrystallized) 77–79 °C; **^1^H NMR** (400 MHz, methanol‐*d*_4_) δ 3.92 (d, *J *= 13.5 Hz, 1H; CH_2_), 3.89 (d, *J* = 13.5 Hz, 1H; CH_2_), 3.75 (d, *J *= 15.4 Hz, 1H; CH_2_), 3.72 (br d, *J* = 15.4 Hz, 1H; CH_2_), 1.55 (q, *J* = 6.5 Hz, 1H; CH), 1.21 (d, *J* = 6.6 Hz, 3H; CH_3_) ppm; **^13^C{^1^H} NMR** (101 MHz, methanol‐*d*_4_) δ 66.9 (CH_2_), 61.2 (CH_2_), 43.1 (CBr_2_), 37.9 (C), 33.2 (CH), 12.0 (CH_3_) ppm; **IR** (neat): 3281 (br), 2936 (w), 1402 (m), 1034 (m), 1019 (s), 646 (m) cm^−1^; **HRMS** (ESI+): *m/z* calcd for C_6_H_10_^79^Br_2_NaO_2_ 294.8940 [*M *+ Na]^+^, found 294.8937 (‐1.0 ppm error).

Characterization of **29** (see Supporting Information for the synthesis of **29**): (*cis/trans* 70:30): ***R*_f_** = 0.22 (DCM/MeOH 97:3); **^1^H NMR** (500 MHz, methanol‐*d*_4_) δ 3.83 (br d, *J* = 11.4 Hz, 1H; CHHOH (*trans*)), 3.81 (d, *J* = 11.7 Hz, 1H; CHHOH (*cis*)), 3.78 (d, *J* = 11.5 Hz, 1H; CHHOH (*trans*)), 3.71 (d, *J* = 11.7 Hz, 1H; CHHOH (*cis*)), 3.69 (d, *J* = 11.9 Hz, 1H; CHHOH (*trans*)), 3.66 (d, *J* = 11.5 Hz, 1H; CHHOH (*cis*)), 3.57 (d, *J* = 11.7 Hz, 1H; CHHOH (*trans*)), 3.41 (d, *J* = 11.4 Hz, 1H; CHHOH (*cis*)), 3.19 (d, *J* = 7.5 Hz, 1H; CHBr (*cis*)), 2.88–2.82 (m, 1H; CHBr (*trans*)), 1.20–1.14 (m, 6H; CH_3_ (*cis* and *trans*)), 1.13–1.06 (m, 1H; CH (*cis* and *trans*)) ppm; **^13^C{^1^H} NMR** (101 MHz, methanol‐*d*_4_) δ 66.34 (CH_2_OH, *cis*), 66.32 (CH_2_OH, *trans*), 61.4 (CH_2_OH, *trans*), 61.3 (CH_2_OH, *cis*), 33.7 (C, *trans*), 33.3 (CHBr, *cis*), 32.3 (CHBr, *trans*), 30.5 (C, *cis*), 27.4 (CH_3_, *trans*), 19.4 (CH_3_, *cis*), 12.5 (CH, *trans*), 10.5 (CH, *cis*) ppm; **IR** (neat): 3316 (br), 2922 (w), 2880 (w), 1408 (w), 1280 (w), 1234 (w), 1018 (s) cm^−1^; **HRMS** (ESI‐): *m/z* calcd for C_6_H_11_^79^Br^35^ClO_2_ 228.9636 [*M *+ Cl]^−^, found 228.9633 (‐1.3 ppm error).

Characterization of **30** (see Supporting Information for the synthesis of **30**): *R*_f _= 0.22 (DCM/MeOH 97:3); **^1^H NMR** (500 MHz, methanol‐*d*_4_) δ 5.32–5.22 (m, 1H; CH), 4.12 (d, *J* = 13.4 Hz, 1H; CHHOH), 4.11 (d, *J* = 12.6 Hz, 1H; CHHOH’), 4.09 (d, *J* = 12.6 Hz, 1H; CHHOH’), 4.08 (d, *J* = 13.4 Hz, 1H; CHHOH), 1.68 (d, *J* = 7.0 Hz, 3H; CH_3_) ppm; **^13^C{^1^H} NMR** (101 MHz, methanol‐*d*_4_) δ 203.3 (C), 105.3 (C), 88.2 (CH), 61.9 (2 × CH_2_), 14.7 (CH_3_) ppm; **IR** (neat): 3300 (br), 2928 (w), 2874 (w), 1140 (w), 1414 (w), 1000 (s) cm^−1^; **HRMS** (ESI+): *m/z* calcd for C_6_H_12_NaO_2_ 139.07405 [*M *+ Na]^+^, found 139.0729 (‐0.4 ppm error).

Characterization of **31** (see Supporting Information for the synthesis of **31**): ***R*_f_** = 0.22 (DCM/MeOH 97:3); **^1^H NMR** (500 MHz, methanol‐*d*_4_) δ 3.78 (d, *J* = 11.4 Hz, 1H; CHHOH), 3.55 (br d, *J* = 11.2 Hz, 1H; CHHOH’), 3.54 (d, *J* = 11.4 Hz, 1H; CHHOH), 3.34 (d, *J* = 11.2 Hz, 1H; CHHOH’), 1.13 (d, *J* = 6.4 Hz, 3H; CH_3_), 0.84 (appdquin, *J* = 8.4, 6.4 Hz, 1H, CHCH_3_), 0.60 (dd, *J* = 8.5, 4.6 Hz, 1H; CHH), 0.13 (appt, *J* = 5.1 Hz, 1H, CHH) ppm; **^13^C{^1^H} NMR** (101 MHz, methanol‐*d*_4_) δ 68.4 (CH_2_OH’), 63.1 (CH_2_OH), 29.2 (C), 16.9 (CH), 16.5 (CH_2_), 14.0 (CH_3_) ppm; **IR** (neat): 3300 (br), 2928 (w), 2874 (w), 1140 (w), 1414 (w), 1000 (s) cm^−1^; **LRMS** (ESI+): 132.1 (100) [*M *+ NH_4_]^+^, **HRMS**: we were unable to obtain an HRMS spectrum.

#### 4.2.6 Synthesis of 1,1‐dibromo‐2‐methyl‐5,7‐dioxa‐6‐thiaspiro[2.5]Octane‐6‐oxide **32**

To a flame‐dried flask under an inert atmosphere containing **28** (30 mg, 0.11 mmol, 1.0 equiv.) was added anhydrous DCM (2 mL). Upon dissolution, the reaction was cooled to 0 °C, and pyridine (0.5 mL) was added. At the same temperature, thionyl chloride (0.02 mL, 0.27 mmol, 2.5 equiv.) was added dropwise. After stirring for 2 hours at room temperature, H_2_O (1 mL) was added and the reaction mixture was stirred for 30 minutes The phases were separated, and the aqueous phase was extracted with DCM (3 × 5 mL). The combined organic layers were dried with MgSO_4_, filtered, and concentrated. Purification through flash column chromatography (SiO_2_, *n*‐pentane/EtOAc 85:15 to 65:35) afforded one of the diastereoisomers of **32** (25 mg, 0.08 mmol, 73%) as a white solid. ***R*_f_** = 0.36 (*n*‐hexane/EtOAc 80:20); **m.p**. (not recrystallized): 73–74 °C; **^1^H NMR** (400 MHz, CDCl_3_) δ 5.17 (br d, *J* = 12.1 Hz, 1H; CHH), 5.06 (br d, *J* = 12.1 Hz, 1H; CHH’), 3.94 (br d, *J* = 12.2 Hz, 1H; CHH), 3.88 (br d, *J* = 12.2 Hz, 1H, CHH), 1.49 (q, *J* = 6.3 Hz, 1H; CH), 1.17 (br d, *J* = 6.4 Hz, 3H; CH_3_) ppm; **^13^C{^1^H} NMR** (101 MHz, CDCl_3_) δ 62.5 (CH_2_), 59.3 (CH_2_), 40.2 (CBr_2_), 32.7 (CH), 30.6 (C), 10.3 (CH_3_) ppm; IR (neat): 2945 (w), 1196 (s), 1182 (s), 964 (s), 671 (s) cm^−1^; **HRMS** (ESI+): *m/z* calcd for C_6_H_8_^79^Br_2_NaO_3_S 340.8453 [*M *+ Na]^+^, found 340.8455 (0.3 ppm error).

Characterization **33** (see Supporting Information for the synthesis of **33**): ***R*_f _** = 0.31 (*n*‐hexane/EtOAc 50:50); **m.p**. (not recrystallized): 129–130 °C; **^1^H NMR** (500 MHz, CDCl_3_) δ 4.64 (d, *J* = 11.4 Hz, 1H; CHH), 4.55 (d, *J* = 11.7 Hz, 1H; CHH’) 4.31 (dd, *J* = 11.5, 1.4 Hz, 1H; CHH), 4.27 (dd, *J* = 11.5, 1.4 Hz, 1H, CHH’), 1.89 (q, *J* = 6.6 Hz, 1H, CH), 1.31 (d, *J* = 6.6 Hz, 3H; CH_3_) ppm; **^13^C{^1^H} NMR** (101 MHz, CDCl_3_) δ 148.3 (CO), 74.1 (CH_2_), 68.9 (CH_2_’), 35.3 (CBr_2_), 33.4 (CH), 27.6 (C), 11.7 (CH_3_) ppm; **IR** (neat): 2989 (w), 2340 (w), 1740 (s), 1169 (s), 1081 (s), 757 (s) cm^−1^; **HRMS** (ESI+) *m/z* calcd for C_7_H_8_^79^Br_2_NaO_3_ 320.8732 [*M *+ Na]^+^, found 320.8735 (0.9 ppm error).

#### 4.2.7 Synthesis of (2‐methylbicyclo[1.1.1]pentan‐1‐yl)(phenyl)Sulfane **34a**

To an oven‐dried flask under argon was added a solution of **23a** (109.5 mg, 2.38 mmol, 1.0 equiv.) in Et_2_O. The flask was cooled to 0 °C, and thiophenol (0.07 mL, 0.69 mmol, 1.5 equiv.) was added. The reaction was then warmed to r.t. and stirred for 1.5 hours. The reaction mixture was washed with 1 M NaOH solution (3 × 5 mL). The organic phase was dried with Na_2_SO_4_, filtered, and concentrated to afford **34a** (438 mg, 2.30 mmol, 98%) as a colorless liquid, with traces of Et_2_O. ***R*_f_** = 0.79 (cyclohexane/Et_2_O 80:20), **^1^H NMR** (400 MHz, CDCl_3_): δ 7.44 (m, 2H; 2 x H_Ar_), 7.29 (m, 3H; 3 x H_Ar_), 2.57 (s, 1H; CH), 2.44 (dd, *J *= 9.7, 2.7 Hz, 1H; H^B^), 2.3 (appquin, *J *= 6.3 Hz, 1H; H^A^), 1.95 (d, *J *= 1.6 Hz, 1H; H^D^), 1.92 (dd, *J *= 6.3, 2.7 Hz, 1H; H^C^), 1.72 (dd, *J *= 9.7, 1.6 Hz, 1H; H^E^), 1.11 (d, *J *= 6.4 Hz, 3H; CH_3_) ppm; **^13^C{^1^H} NMR** (101 MHz, CDCl_3_): δ 133.8 (2 x CH_Ar_), 133.6 (C_Ar_), 128.6 (2 x CH_Ar_), 127.4 (CH_Ar_), 58.5 (C_2_), 53.5 (C_5_), 48.8 (C_4_), 48.2 (C_1_), 32.3 (C_3_), 10.1 (CH_3_) ppm; **IR** (neat) 2972 (m), 2927 (m), 2895 (m), 691 (s) cm^−1^; **HRMS** (EI) *m/z* calcd for C_12_H_14_S 190.0811 [*M*]^+.^, found 190.0809 (1.00 ppm error).

#### 4.2.8 Synthesis of Methyl 3‐((2‐methylbicyclo[1.1.1]pentan‐1‐yl)thio)Propanoate **35a**

To a flame‐dried flask under an argon atmosphere was added a solution of **23a** (95 mg, 1.18 mmol, 1.0 equiv.) in Et_2_O. The flask was cooled to 0 °C, and methyl 3‐mercaptopropionate (0.2 mL, 1.8 mmol, 1.5 equiv.) was added. The reaction was warmed to r.t. and stirred for 1.5 hours. The reaction was washed with 1 M NaOH solution (3 ×10 mL). The organic phase was dried with Na_2_SO_4_, filtered, and concentrated. Purification through flash column chromatography (SiO_2_, 100% *n*‐pentane) afforded **35a** (210 mg, 1.0 mmol, 90%) as a colorless liquid. ***R*_f_** = 0.42 (*n*‐hexane/Et_2_O 90:10); **^1^H NMR** (400 MHz, CDCl_3_): δ 3.70 (s, 3H; OCH_3_), 2.79–2.71 (m, 2H; SCH_2_), 2.62–2.56 (m, 2H; CH_2_), 2.58 (s, 1H; CH), 2.43 (dd, *J *= 9.7, 2.8 Hz, 1H; H^B^), 2.35 (appquin, *J* = 6.3 Hz, 1H; H^A^), 1.96 (dd, *J* = 6.3, 2.8 Hz, 1H; H^C^), 1.78 (dd, *J* = 9.7, 1.7 Hz, 1H; H^E^), 1.15 (d, *J* = 6.4 Hz, 3H; CH_3_) ppm; **^13^C{^1^H} NMR** (101 MHz, CDCl_3_): δ 172.3 (CO), 58.1 (CH; C_2_), 53.1 (CH_2_; C_5_), 51.7 (OCH_3_), 48.5 (CH_2_; C_4_), 46.9 (C; C3), 35.5 (CH_2_), 32.4 (CH), 25.4 (SCH_2_), 10.1 (CH_3_) ppm; **IR** (neat): 2971 (m), 2928 (m), 1737 (s), 1436 (m) cm^−1^; **HRMS** (ESI+): *m/z* calcd for C_10_H_16_NaO_2_S 223.0763 [*M *+ Na]^+^, found 223.0759 (1.8 ppm error).

#### 4.2.9 Synthesis of *N*,*N*‐dibenzyl‐2‐methylbicyclo[1.1.1]Pentan‐1‐amine **36a**

To a flame‐dried pressure tube under argon atmosphere was added **22** (90 mg, 0.29 mmol, 1.0 equiv.) and anhydrous Et_2_O (2 mL). The solution was cooled to −45 °C, and PhLi (0.3 mL, 1.9 M in Bu_2_O, 0.57 mmol, 2.0 equiv.) was added slowly via syringe. The reaction was stirred for 5 minutes, warmed to 0 °C, and stirred for 2 hours. The reaction was allowed to warm to room temperature, and a freshly prepared ethereal solution of Bn_2_NMgCl•LiCl (0.74 mL, 3.2 mmol, 11.0 equiv.) was slowly added. After the addition, the septum was replaced with a screw cap, and the reaction was transferred to a preheated oil bath at 50 °C. After stirring for 16 hours at 50 °C, the reaction was cooled to 0 °C and quenched slowly with sat. aq. NH_4_Cl (3 mL). The mixture was then diluted with EtOAc (5 mL), the phases were separated, and the aqueous layer was extracted with EtOAc (3 × 5 mL). The combined organic phases were dried with MgSO_4_, filtered, and concentrated. Purification through flash column chromatography (SiO_2_, *n*‐hexane/EtOAc 80:20) afforded **36a** (22 mg, 0.08 mmol, 27%) as a light‐yellow liquid. ***R*_f_** = 0.58 (*n*‐hexane/EtOAc 98:2); **^1^H NMR** (400 MHz, CDCl_3_) δ 7.34–7.39 (m, 4H; 4 × Ar─H), 7.23–7.30 (m, 4H; 4 × Ar─H), 7.16–7.22 (m, 2H; 2 × Ar─H), 3.65 (d, *J* = 13.7 Hz, 2H; 2 × NCHH), 3.59 (d, *J* = 14.3 Hz, 2H; 2 × NCHH), 2.20 (appquin, *J* = 6.3 Hz, 1H; H^A^), 2.13 (dd, *J* = 9.7, 2.8 Hz, 1H; H^B^), 2.10 (s, 1H; CH), 1.70 (d, *J* = 1.5 Hz, 1H; H^D^), 1.61 (dd, *J* = 6.4, 2.8 Hz, 1H; H^C^), 1.50 (dd, *J *= 9.7, 1.5 Hz, 1H; H^E^), 1.00 (d, *J *= 6.2 Hz, 1H; CH_3_) ppm; **^13^C{^1^H} NMR** (101 MHz, CDCl_3_) δ 140.8 (2C; 2 × C_Ar_), 128.4 (4C; 4 × CH_Ar_), 127.9 (4C; 4 × CH_Ar_), 126.5 (2C; 2 × CH_Ar_), 63.0 (CN), 55.5 (C_2_), 54.7 (2C; 2 × CH_2_N), 47.0 (C_5_), 46.0 (C_4_), 26.7 (CH), 10.2 (CH_3_) ppm; **IR** (neat): 2963(m), 1452 (m), 739 (s), 696 (s) cm^−1^; **HRMS** (ESI+): *m/z* calcd for C_20_H_23_N 278.1903 [*M *+ H]^+^, found 278.1907 (1.4 ppm error).

#### 4.2.10 Synthesis of 1‐1′‐(2‐methylbicyclo[1.1.1]pentane‐1,3‐diyl)bis(ethan‐1‐one) **37a**

To a solution of **23a** (172.6 mg, 2.1 mmol, 1.0 equiv.) in anhydrous Et_2_O under an inert atmosphere was added 2,3 butanedione (0.28 ml, 3.15 mmol, 1.5 equiv.) at −40 °C. The solution was allowed to warm to room temperature, degassed with He, and pumped into a photoreactor (365 nm, 12 W, 4 mL) at a rate of 2.8 ml/min. The resulting solution was concentrated and purified through flash chromatography (SiO_2_, pentane/MTBE 85:15 to 50:50) to afford **37a** (307 mg, 1.85 mmol, 88%) as a colorless liquid. ***R*_f_** = 0.30 (cyclohexane/MTBE 50:50); **^1^H NMR** (400 MHz, CDCl_3_) δ 2.78 (appquin, *J* = 6.2 Hz, 1H; H^A^), 2.75 (dd, *J *= 9.6, 3.0 Hz, 1H; H^B^), 2.22 (d, *J* = 1.5 Hz; H^D^), 2.18 (dd, *J* = 6.3, 3.1 Hz; H^C^), 2.10 (s, 6H; 2 × OCH_3_), 2.11 (dd, *J* = 9.4, 1.8 Hz; H^E^), 1.28 (d, *J* = 6.2 Hz, 3H; CH_3_) ppm; **^13^C{^1^H} NMR** (101 MHz, CDCl_3_) δ 205.33 (2C; 2 × CO), 59.24 (C_2_), 52.09 (C_5_), 45.99 (2C; C_1_, C_3_), 45.30 (C_4_), 26.56 (2C; 2 × COCH_3_), 9.72 (CH_3_) ppm; **IR** (neat): 2998 (m), 2935 (m), 1696 (s), 1392 (m) cm^−1^; **HRMS** (ESI+): *m/z* calcd for C_10_H_15_O_2_ 167.1067 [*M *+ H]^+^, found 167.1070 (1.8 ppm error).

#### 4.2.11 Synthesis of Ethyl 2,2‐difluoro‐2‐(3‐iodo‐2‐methylbicyclo[1.1.1]pentan‐1‐yl)acetate **38a**

To a flame‐dried flask under an argon atmosphere was added, a solution of **23a** (37 mg, 0.46 mmol, 1.0 equiv.) in Et_2_O followed by ethyl iododifluoroacetate (0.08 mL, 0.55 mmol, 1.2 equiv.). The solution was cooled to 0 °C and BEt_3_ (1 M in *n‐*hexane, 1 drop, catalytic amount) was added. The reaction was stirred at the same temperature for 45 minutes and the solvents evaporated. Purification through flash column chromatography (SiO_2_, *n*‐hexane/EtOAc 99:1) afforded **38a** (114 mg, 0.3 mmol, 75%) as a colorless liquid. ***R*_f_** = 0.22 (*n*‐hexane/EtOAc 98:2); **^1^H NMR** (400 MHz, CDCl_3_) δ 4.34 (dq, *J* = 10.9, 7.1 Hz, 1H; OCHH), 4.33 (dq, *J* = 10.9, 7.1 Hz, 1H; OCHH), 2.86 (dd, *J* = 9.5, 2.9 Hz, 1H; H^B^), 2.69 (appquin, *J* = 6.3 Hz, 1H; H^A^), 2.44 (d, *J *= 2.0 Hz, 1H; H^D^), 2.42 (dd, *J* = 6.4, 2.9 Hz, 1H; H^C^), 2.20 (dd, *J* = 9.5, 1.8 Hz, 1H; H^E^), 1.36 (t, *J *= 7.1 Hz, 3H; OCH_2_CH_3_), 1.13 (d, *J* = 6.4 Hz, 3H; CH_3_) ppm; **^19^F NMR** (376 MHz, CDCl_3_) δ −108.2 (d, *J* = 263.6 Hz, 1F; CFF), −109.0 (d, *J* = 263.6 Hz, 1F; CFF) ppm; **^13^C{^1^H} NMR** (101 MHz, CDCl_3_) δ 162.6 (t, *J *= 33.0 Hz, CO), 110.8 (t, *J* = 252.4 Hz; CF_2_), 63.2 (t, *J *= 2.6 Hz; C_2_), 63.0 (OCH_2_), 56.8 (t, *J* = 3.3 Hz; C_5_), 53.1 (t, *J* = 2.9 Hz; C_4_), 47.4 (t, *J* = 31.5 Hz; CCF_2_), 14.0 (OCH_2_ CH_3_), 12.1 (t, *J* = 2.2 Hz; CI), 9.9 (CH_3_) ppm; **IR** (neat): 2985 (m), 1764 (s), 1301 (s), 1195 (s), 1151 (s), 1103 (s) cm^−1^; **HRMS** (EI): *m/z* calcd for C_10_H_13_F_2_O_2_ 203.0878 [*M*‐I]^+•^, found 203.0880 (0.98 ppm error).

#### 4.2.12 Synthesis of 2‐(3‐ethylidene‐1‐iodocyclobutyl)acetamide **40**

To a flame‐dried flask under argon was added a solution of **23a** (0.15 M in Et_2_O, 5 mL, 56 mg, 0.70 mmol, 1.3 equiv.) and cooled to 0 °C. A solution of iodoacetamide (100 mg, 0.54 mmol, 1.0 equiv.) in anhydrous MeOH (0.5 mL), followed by BEt_3_ (1 M in *n*‐hexane, 0.05 mL, 10 mol%). The reaction was stirred at 0 °C for 5 minutes and warmed to room temperature. After stirring for 1.5 hours, the solvent was evaporated under reduced pressure. Purification through two successive flash chromatographies (SiO_2_, 100% EtOAc and *n*‐hexane/EtOAc 65:35) afforded **40** (43 mg, 0.16 mmol, 30%) as a colorless semisolid. ***R*_f_** = 0.19 (100% EtOAc); **^1^H NMR** (400 MHz, CDCl_3_) δ 5.57 (br s, 1H; NHH), 5.42 (br s, 1H; NHH), 5.32 (qquin, *J* = 6.7, 2.3 Hz, 1H; CH), 3.25–3.55 (m, 4H; 2 × CH_2_), 2.95 (s, 2H; CH_2_CO), 1.51 (dq, *J* = 8.6, 1.9 Hz, 3H, CH_3_) ppm; **^13^C{^1^H} NMR** (101 MHz, CDCl_3_) δ 186.7 (CO), 118.7 (CH), 52.9 (CH_2_), 42.1 (CH_2_CO), 51.1 (CH_2_), 32.0 (C), 13.6 (CH_3_), 9.6 (CI) ppm; **IR** (neat) 3380 (br), 3196 (br), 2911 (w), 1665 (s), 1231 (m) cm^−1^; MS (ESI+) *m/z* 288.1 [*M *+ Na]^+^; **HRMS** (ESI+): *m/z* calcd for C_8_H_12_INNaO 287.9857 [*M *+ Na]^+^, found 287.9856 (‐0.5 ppm error).

#### 4.2.13 Synthesis of 2‐(2‐methylbicyclo[1.1.1]pentan‐1‐yl)acetamide **43a**

To a flame‐dried flask under inert atmosphere was added a solution of **23a** (0.15 M in Et_2_O, 20 mL, 264 mg, 3.3 mmol, 1.0 equiv.) and was cooled to 0 °C. A solution of iodoacetamide (607 mg, 3.3 mmol, 1.0 equiv.) in anhydrous MeOH (1.5 mL) was added at 0 °C, followed by (1 M solution in *n*‐hexane, 0.05 mL, 10 mol%). The reaction was stirred 5 minutes at 0 °C and warmed to room temperature. After stirring for 2 hours, TTMSS (1.3 mL, 4.3 mmol, 1.3 equiv.) and BEt_3_ (1 M solution in *n*‐hexane, 0.35 mL, 10 mol%) were added, and the reaction was stirred for 2 hours at room temperature. The solvent was evaporated under reduced pressure, and purification through flash chromatography (SiO_2_, 100% EtOAc) and RP‐HPLC (C_18_ silica, gradient 25% to 45% aq. ACN (0.1% TFA), 20 minutes) afforded **43a** (139 mg, 1.0 mmol, 30%) as white fine crystals. ***R*_f_** = 0.39 (EtOAc); **m.p**. (not recrystallized) 118–120 °C, **^1^H NMR** (500 MHz, CDCl_3_) δ 5.31 (br s, 2H; NHH, NHH), 2.35 (s, 1H; CH), 2.33 (dd, *J* = 9.9, 2.7 Hz, 1H; H^B^), 2.30 (s, 2H; CH_2_), 2.21 (appquin, *J* = 6.3 Hz, 1H; H^A^), 1.82 (d, *J* = 1.3 Hz,1H; H^D^), 1.77 (dd, *J* = 6.4, 2.7 Hz, 1H; H^C^), 1.61 (dd, *J* = 9.8, 1.3 Hz, 1H; H^E^), 1.13 (d, *J* = 6.3 Hz, 3H; CH_3_) ppm; **^13^C{^1^H} NMR** (101 MHz, CDCl_3_) δ 173.0 (CO); 55.7 (C_2_); 50.5 (C_5_); 46.1 (C_4_); 43.5 (C_1_); 38.5 (CH_2_); 31.8 (CH); 10.1 (CH_3_) ppm; **IR** (neat) 3356 (m), 3177 (m), 2965 (m), 2891 (w), 1665 (s), 1594 (vs.), 1434 (m), 1406 (s), 1267 (s), 1196 (m), 684 (m), 633 (m) cm^−1^; **LRMS** (ESI+) *m/z* 140.2 (100) [*M *+ H]^+^; **HRMS** (ESI+): *m/z* calcd for C_8_H_13_NNaO 162.0889 [*M *+ Na]^+^, found 162.0890 (‐0.2 ppm error).

#### 4.2.14 Synthesis of Methyl *N*‐(*tert*‐butoxycarbonyl)‐*S*‐((2*R*)‐2‐methylbicyclo[1.1.1]Pentan‐1‐yl)‐l‐cysteinate **(l, r )‐44** and Methyl *N*‐(*tert*‐butoxycarbonyl)‐*S*‐((2*S*)‐2‐methylbicyclo[1.1.1]Pentan‐1‐yl)‐l‐cysteinate **(l,*S*)‐44**

To a flame‐dried flask under argon was added a solution of **23a** (7.3 mg, 0.09 mmol, 1.0 equiv.) in Et_2_O. The flask was cooled to 0 °C and *N*‐(tert‐butoxycarbonyl)‐l‐cysteine methyl ester (0.02 mL, 0.12 mmol, 1.3 equiv.) was added. The reaction was then warmed to r.t. and stirred for 1.5 hours. The reaction mixture was washed with 1 M NaOH solution (3 × 5 mL). The organic phase was dried with Na_2_SO_4_, filtered, and concentrated. Purification through flash chromatography (*n*‐pentane:Et_2_O 4:1) afforded **44** as a 1:1 mixture of diastereoisomers (32.7 mg, 0.08 mmol, 91%) as a colorless liquid. ***R*_f_** = 0.38 (*n*‐hexane/Et_2_O 80:20); **^1^H NMR** (400 MHz, CDCl_3_) δ 5.28 (br s, 2 × 1H; NH, NH’), 4.52 (t, *J* = 4.9 Hz, 1H, NHCH), 4.51 (t, *J *= 5.1 Hz, 1H; NHCH’), 3.76 (s, 6H; 3 × OCH_3_, 3 × OCH’_3_), 2.85–2.97 (m, 4H; SCH_2_, SCH_2_’), 2.57 (s, 2H; CH, CH’), 2.40 (dd, *J* = 9.7, 2.7 Hz, 1H; H^B^), 2.39 (dd, *J* = 9.8, 2.7 Hz, 1H; H^B^’), 2.32 (appquint, *J *= 6.3 Hz, 2H; H^A^, H^A^’), 1.93 (d, *J* = 1.7 Hz, 1H; H^D^), 1.92 (d, *J* = 1.7 Hz, 1H; H^D^’),1.89–1.91 (m, 2H; HC, HC’), 1.76 (dd, *J* = 9.8, 1.7 Hz, 1H; H^E^), 1.75 (dd, *J* = 9.8, 1.7 Hz, 1H; H^E^’), 1.45 (s, 18H; CH(CH_3_)_3_, CH(CH’_3_)_3_), 1.13 (br d, *J* = 6.4 Hz, CH_3_), 1.13 (d, *J *= 6.4 Hz, CH’_3_) ppm; **^13^C{^1^H} NMR** ((101 MHz, CDCl_3_) δ 171.4 (CO), 171.4 (C'O), 155.0 (2C; NCO, NC'O), 80.0 (2C; CHCH_3_, CH'CH_3)_ 58.1 (C_2_), 58.0 (C’_2_), 53.4 (CHNH), 53.4 (C'HNH), 53.1 (C_5_), 53.0 (C_5_’), 52.5 (2C; OCH_3_, OC'H_3_), 48.4 (C_4_), 48.4 (C’_4_), 46.8 (CS), 46.8 (C'S), 32.9 (SCH_2_), 32.9 (SC'H_2_), 32.0 (2C; CH, C'H), 28.3(6C; CH(CH_3_)_3_, CH(C’H_3_)_3_), 10.0 (CH_3_), 10.0 (C'H_3_) ppm; **IR** (neat): 3437 (w), 3367 (w), 2975 (m), 2930 (m), 1749 (s), 1734 (s), 1206 (m), 1157 (s) cm^−1^; **HRMS** (ESI+): *m/z* calcd for C_15_H_25_NNaO_4_S 338.1396 [*M *+ Na]^+^; found 338.1396 (0.1 ppm error).

#### 4.2.15 Synthesis of (2‐methylbicyclo[1.1.1]pentanyl)(phenyl)Sulfoximine **45**

Thioether **34a** (64 mg, 0.33 mmol, 1.0 equiv.) was dissolved in MeOH (3 mL) at r.t. (NH_4_)_2_CO_3_ (68 mg, 0.7 mmol, 2.1 equiv.) was added, followed by PhI(OAc)_2_ (330.8 mg, 1.0 mmol, 3.0 equiv.). The reaction mixture was stirred for 30 minutes, followed by removal of the solvent under reduced pressure. Purification through flash chromatography (SiO_2_, *n*‐hexane:EtOAc 85:15) afforded an inseparable mixture of diastereoisomers **45** (17 mg, 0.07 mmol, 23%) as a viscous oil. ***R*_f_ **= 0.26 (*n*‐hexane/EtOAc 65:35); **^1^H NMR** (400 MHz, CDCl_3_) δ 7.89–7.95 (m, 4H, 2 × Ar─H, 2 × Ar─ H’), 7.58–7.64 (m, 2H; Ar─H, Ar─H’), 7.50–7.56 (m, 4H; 2 × Ar─H; 2 × Ar─H’), 2.62 (dd, *J* = 9.5, 2.6 Hz, 1H; H^B^’), 2.61 (dd, *J* = 9.5, 2.6 Hz, 1H; H^B^), 2.52 (s, 1H; CH’), 2.52 (s, 1H; CH’), 2.49 (appquin, *J* = 6.4 Hz, 1H; H^A^), 2.45 (appquin, *J* = 6.3 Hz, 1H; H^A^’), 2.09 (dd, *J* = 6.3, 2.9 Hz, 1H; H^C^), 2.04 (dd, *J* = 6.2, 2.6 Hz, 1H; H^C’^), 2.04 (d, *J *= 2.0 Hz, 1H; H^D^), 2.04 (d, *J* = 1.6 Hz, 1H; H^D^’), 1.77 (dd, *J* = 9.6, 1.9 Hz, 1H; H^E^), 1.78 (dd, *J* = 9.5, 1.8 Hz, 1H; H^E^’), 1.14 (d, *J* = 6.2 Hz, 3H; CH_3_), 1.14 (d, *J* = 6.2 Hz, 3H; CH’_3_) ppm; **^13^C{^1^H} NMR** (101 MHz, CDCl_3_) δ 139.9 (C_Ar_), 139.7 (C’_Ar_), 132.9 (4C; 2 × CH_Ar_, 2 × C'H_Ar_), 128.9 (4C; 2 × C'H_Ar_, 2 × CH_Ar_), 128.9 (CH_Ar_), 128.8 (CH’_Ar_), 57.7 (C’), 57.6 (C), 57.5 (C’_2_), 57.3 (C_2_), 50.1 (C’_5_), 49.9 (C_5_), 45.4 (C_4_), 45.4 (C’_4_), 29.8 (2C; CH, C'H), 10.3 (CH_3_), 10.2 (C'H_3_) ppm; **IR** (neat): 3261 (w), 2976 (w), 2933 (w), 1445 (m), 1218 9 (s), 1202 (s) cm^−1^; **HRMS** (ESI+): *m/z* calcd for C_12_H_15_NNaOS 244.0767 [*M *+ Na]^+^; found 244.0768 (0.4 ppm error).

#### 4.2.16 Synthesis of 2,2‐difluoro‐2‐(2‐methylbicyclo[1.1.1]pentan‐1‐yl)ethan‐1‐ol **46a**

To a flame‐dried flask under inert atmosphere was added **38a** (385 mg, 1.17 mmol, 1.0 equiv.) in anhydrous EtOH (5 mL). TTMSS (0.43 mL, 1.4 mmol, 1.2 equiv.) was added, and BEt_3_ (1 M in *n*‐hexane, 0.01 mL, 1 mol%) was added via syringe. The reaction stirred 2 hours at r.t. The reaction was cooled to 0 °C, and LiAlH_4_ (1 M solution in Et_2_O, 0.58 mL, 1.17 mmol, 1.0 equiv.) was added dropwise. The reaction was warmed to r.t. After 2 hours, it was cooled to 0 °C, and H_2_O (5 mL) was added dropwise. The reaction was acidified with 1 M HCl solution to pH 3, the phases separated, and the aqueous phase extracted with Et_2_O (3 × 5 mL). The combined organic phases were dried with Na_2_SO_4_, filtered, and concentrated. Purification through flash chromatography (SiO_2_, 100% pentane to *n*‐pentane:Et_2_O 80:20) afforded **46a** (64 mg, 0.34 mmol, 29%) as a clear colorless volatile liquid. ***R*_f_** = 0.39 (*n*‐pentane/Et_2_O 65:35); **^1^H NMR** (400 MHz, CD_2_Cl_2_) δ 3.69 (t, *J* = 13.8 Hz, 2H; CH_2_CF_2_), 2.49 (dd, *J* = 9.8, 2.9 Hz, 1H; H^B^), 2.41 (appquin, *J* = 6.3 Hz, 1H; H^A^), 2.32 (s, 1H, CH), 1.89 (dd, *J* = 6.6, 2.9 Hz, 1H; H^C^), 1.89 (d, *J* = 2.1 Hz, 1H; H^D^), 1.71 (br s, 1H, OH), 1.68 (ddt, *J* = 9.8, 1.7, 0.5 (2 ×) Hz, 1H; H^E^), 1.19 (dt, *J* = 6.4, 0.8 (2 ×), 3H; CH_3_) ppm; **^19^F NMR** (376 MHz, CD_2_Cl_2_) δ −114.5 (br dt, *J* = 256.6, 13.9 Hz, 1F, CFF), −115.2 (br dt, *J* = 258.4, 13.0 Hz, 1F; CFF) ppm; **^19^F{^1^H} NMR** (376 MHz, CD_2_Cl_2_) δ −144.5 (d, *J* = 257.5 Hz, 1F; CFF), −115.2 (d, *J* = 256.6 Hz, 1F; CFF) ppm; ^13^C{^1^H} NMR (101 MHz, CD_2_Cl_2_) δ 119.2 (t, *J* = 241.4 Hz; CF_2_), 63.5 (t, *J* = 29.7 Hz; CH_2_OH), 55.8 (t, *J* = 3.3 Hz; C_2_), 48.4 (t, *J* = 4.4 Hz; C_5_), 44.3 (t, *J* = 3.7 Hz; C_4_), 32.0 (t, *J* = 2.2 Hz, CH; C_3_), 11.2 (CH_3_) ppm; NOTE: CCF_2_ not observed; **IR** (neat): 3299 (br), 2955 (w), 2912 (w), 1312(s) cm^−1^; **HRMS** (ESI+): compound decomposed on spectrometer.

#### 4.2.17 Synthesis of 2‐(bicyclo[1.1.1]pentan‐1‐yl)‐2,2‐difluoroethan‐1‐ol **46b**

To a flame‐dried flask under inert atmosphere was added **38b** (1.80 g, 5.70 mmol, 1.0 equiv.) in anhydrous MeOH (6 mL). TTMSS (2.1 mL, 6.80 mmol, 1.2 equiv.) was added, followed by dropwise addition (needle tip in solution) of BEt_3_ (1 M in hexane, 0.6 mL, 10 mol%). The reaction was stirred 2 hours at r.t. and the solvent was carefully evaporated under reduced pressure. ^1^H NMR of the crude reaction mixture indicated quantitative iodide reduction and transesterification to the corresponding methyl ester. The crude reaction mixture was used directly for the next step. Methyl 2‐(bicyclo[1.1.1]pentan‐1‐yl)‐2,2‐difluoroacetate (nominally 5.7 mmol, 1.0 equiv.) was dissolved in Et_2_O (6 mL) and cooled to 0 °C, and LiAlH_4_ (1 M solution in THF, 5.7 mL, 5.70 mmol, 1.0 equiv.) was added dropwise. The reaction was warmed to r.t. and stirred for 2 hours, cooled to 0 °C, and Na_2_SO_4_•10H_2_O was added portion‐wise until hydrogen generation ceased. The reaction mixture was filtered and concentrated under reduced pressure. Purification through flash chromatography (SiO_2_, cyclohexane/Et_2_O 80:20) a **46b** (287 mg, 1.94 mmol, 34%) as a clear volatile liquid. ***R*_f_** = 0.25 (cyclohexane/Et_2_O 65:35); **^1^H NMR** (500 MHz, CDCl_3_) δ 3.76 (t, *J* = 13.3 Hz, 2H; CF_2_CH_2_), 2.53 (s, 1H; CH), 1.98 (s, 1H; OH), 1.95 (s, 6H; 3 × CH_2_(BCP)) ppm; **^19^F NMR** (470 MHz, CDCl_3_) δ −115.6 (br t, *J* = 13.2 Hz; 2F) ppm; **^19^F{^1^H} NMR** (470 MHz, CDCl_3_) δ −115.6 (s; 2F) ppm; **^13^C{^1^H} NMR** (101 MHz, CDCl_3_) δ 117.9 (t, *J* = 242.3 Hz; CF_2_), 62.9 (t, *J* = 29.4 Hz; CH_2_CF_2_), 48.7 (t, *J* = 3.7 Hz, 3C; 3 × CH_2_(BCP)), 43.3 (t, *J* = 31.6 Hz; C), 27.5 (t, *J* = 2.0 Hz; CH) ppm; **IR** (neat): 3358 (br), 2928 (w), 2920 (w), 2884 (w) cm^−1^; **HRMS** (ESI+): compound decomposed on spectrometer.

#### 4.2.18 Synthesis of 2,2‐difluoro‐2‐(*o*‐tolyl)ethan‐1‐ol **47a**

To a flame‐dried flask under an argon atmosphere was added a solution of LiAlH_4_ in THF (1 M, 3.36 mL, 1.2 equiv.). The solution was cooled to 0 °C, and a solution of **54a** (600 mg, 2.80 mmol, 1.0 equiv.) in anhydrous THF (3 mL) was added dropwise. The reaction was left to stir overnight at r.t. The reaction mixture was cooled to 0 °C, and under a gentle flow of argon, Na_2_SO_4_•10H_2_O was carefully added until hydrogen generation ceased. The solution was filtered and concentrated under reduced pressure. Purification through flash column chromatography (SiO_2_, *n*‐pentane/Et_2_O 80:20) afforded **47a** (384 mg, 2.23 mmol, 80%) as a colorless liquid. ***R*_f_** = 0.34 (cyclohexane/Et_2_O 70:30); **^1^H NMR** (400 MHz, CDCl_3_) δ 7.46–7.53 (m, 1H; Ar─H), 7.33–7.38 (m, 1H; Ar─H), 7.22–7.29 (m, 2H; 2 × Ar─H), 4.02 (td, *J* = 13.8, 7.2 Hz, 2H; CH_2_), 2.48 (appt, *J *= 2.6 Hz, 3H; CH_3_), 2.11 (t, *J* = 7.2 Hz, 1H; OH) ppm; **^19^F NMR** (470 MHz, CDCl_3_) δ −104.1 (br t, *J* = 13.6 Hz, 2F) ppm; **^19^F{^1^H} NMR** (470 MHz, CDCl_3_) δ −104.1 (s, 2F) ppm; **^13^C{^1^H} NMR** (101 MHz, CDCl_3_) δ 136.1 (br t, *J* = 2.7 Hz, C_Ar_; C_2_), 132.1 (t, *J* = 24.0 Hz, C_Ar_; C_1_), 132.1 (CH_Ar_; C_4_), 130.3 (t, *J* = 1.5 Hz, CH_Ar_; C_3_), 126.7 (t, *J* = 8.7 Hz, CH_Ar_; C_6_), 125.8 (CH_Ar;_ C_5_), 121.7 (t, *J* = 241.2 Hz; CF_2_), 65.3 (t, *J *= 31.2 Hz; CH_2_), 20.2 (t, *J* = 3.8 Hz; ArCH_3_) ppm; **IR** (neat) 3300 (br), 2942 (w), 1458 (m), 1309 (m), 1250 (s), 1179 (s), 1069 (s), 1052 (s), 979 (s) cm^−1^; **LRMS** (EI, 70 eV) m/z (%): 172 (20) [*M*]^+^, 141 (100) [M─CH_2_OH]^+^, 91 (15) [C_7_H_7_]^+^; **HRMS** (ESI+): compound decomposed on spectrometer.

#### 4.2.19 Synthesis of 2,2‐difluoro‐2‐phenylethan‐1‐ol **47b**

To a flame‐dried flask under inert atmosphere was added **54b** (1.0 g, 5.0 mmol, 1.0 equiv.) and anhydrous THF (15 mL). The solution was cooled to 0 °C, and LiAlH4 (1 M in THF, 5 mL, 1.0 equiv.) was added dropwise. The reaction was stirred for 5 minutes at 0 °C and allowed to warm to r.t. The reaction was stirred overnight. The reaction was cooled to 0 °C, and Na_2_SO_4_•10H_2_O was added portion‐wise until hydrogen formation ceased. The reaction mixture was filtered, dried over Na_2_SO_4_, filtered, and concentrated under reduced pressure. Purification through flash column chromatography (SiO_2_, *n*‐hexane/Et_2_O 80:20) afforded **47b** (498 mg, 3.1 mmol, 62%) as a colorless liquid. ***R*_f _** = 0.23 (*n*‐hexane/EtOAc 90:10), **^1^H NMR** (400 MHz, CDCl_3_) δ 7.49–7.57 (m, 2H; 2 × Ar─H), 7.43–7.50 (m, 3H; 3 × Ar─H), 3.96 (td, *J* = 13.5, 6.6 Hz, 2H; CH_2_OH), 2.31 (t, *J* = 6.8 Hz, 1H; OH) ppm; **^19^F NMR** (470 MHz, CDCl_3_) δ −170.15 (br t, *J* = 13.2 Hz, CF_2_) ppm; **^19^F{^1^H} NMR** (470 MHz, CDCl_3_) δ −170.15 (s, CF_2_) ppm; **^13^C{^1^H} NMR** (101 MHz, CDCl_3_) δ 134.4 (t, *J* = 25.1 Hz, 1C; C_Ar_, C_1_), 130.3 (CH_Ar_; C_4_), 128.5 (2 × CH_Ar_; C_3_, C_5_), 125.4 (t, *J* = 6.2 Hz, 2 × CH_Ar_; C_2_, C_6_), 120.6 (t, *J* = 246.3 Hz; CF_2_), 65.9 (t, *J* = 32.3 Hz; CH_2_) ppm; **IR** (neat) 3343 (br), 1183 (m), 1054 (s), 1001 (s) cm^−1^; **HRMS** (ESI+) compound decomposed on spectrometer.

#### 4.2.20 Synthesis of 2‐methyl‐1‐((4‐nitrophenyl)sulfonyl)Bicyclo[1.1.1]Pentane **49a**

To a flame‐dried flask under argon was added **56a** (15 mg, 0.09 mmol, 1.0 equiv.), 1‐fluoro‐4‐nitrobenzene (0.01 mL, 0.09 mmol, 1.0 equiv.), and anhydrous DMF (1 mL). The reaction was warmed to 80 °C and stirred for 3 days. Purification of the crude mixture through two successive flash chromatographies (SiO_2_, *n*‐hexane:EtOAc 85:15, *n*‐hexane:EtOAc 95:5) afforded **49a** (18 mg, 0.07 mmol, 76%) as a pale‐yellow solid. ***R*_f_** = 0.13 (*n*‐hexane/EtOAc 80:20); **m.p**. (recrystallized from Et_2_O) 91–92 °C; **^1^H NMR** (400 MHz, CDCl_3_) δ 8.40–8.44 (m, 2H; 2 × H_Ar_), 8.03–8.08 (m, 2H; 2 × H_Ar_), 2.72 (dd, *J* = 9.5, 2.9 Hz, 1H; H^B^), 2.60 (s, 1H; CH), 2.56 (appquin, *J* = 6.3 Hz, 1H; H^A^), 2.12 (dd, *J* = 6.3, 3.0 Hz, 1H; H^C^), 2.08 (d, *J* = 2.0 Hz, 1H; H^D^), 1.83 (dd, *J* = 9.7, 2.0 Hz, 1H; H^E^), 1.22 (d, *J* = 6.2 Hz, 3H; CH_3_) ppm; **^13^C{^1^H} NMR** (101 MHz, CDCl_3_) δ 150.9 (C_Ar_NO_2_), 143.5 (C_Ar_S), 129.9 (2C; 2 × C_Ar_), 124.3 (2C; 2 × C_Ar_), 57.9 (C_2_), 56.7 (CS), 50.3 (C_5_), 45.9 (C_4_), 31.2 (CH), 10.4 (CH_3_) ppm; **IR** (neat): 2982 (w), 2928 (w), 1528 (s), 1348 (m), 1293 (s) cm^−1^; **HRMS** (ESI+): *m/z* calcd for C_12_H_13_NNaO_4_S 290.0457 [*M *+ Na]^+^; found 290.0462 (1.7 ppm error).

#### 4.2.21 Synthesis of Ethyl 2,2‐difluoro‐2‐(*o*‐tolyl)acetate **54a**

##### Activation of Cu

Copper powder (1.700 g, 26 mmol, 1.0 equiv.) was stirred vigorously in aq. HCl (1 M, 10 mL) for 10 minutes at r.t. and filtered. This procedure was repeated with water (10 mL), MeOH (10 mL), and acetone (10 mL), respectively. Finally, the copper powder was dried under vacuum for 15 minutes before use.^[^ ^67^ ^]^

**54a** was prepared following a literature procedure.^[^ ^64^ ^]^ The activated copper was suspended in DMSO (26 mL) under an argon atmosphere. 2‐Iodotoluene (1.853 g, 8.5 mmol, 1.0 equiv.) and ethyl bromodifluoroacetate **EBDFA** (1.11 mL, 8.5 mmol, 1.0 equiv.) were added to the suspension. The reaction was stirred at 60 °C for 12 hours, after which it was poured into a mixture of ice and sat. aq. NH_4_Cl (100 mL). The aqueous layer was extracted with EtOAc (3 × 50 mL). The combined organic phases were washed with brine (50 mL), dried over MgSO_4_, filtered, and concentrated under reduced pressure. Purification through flash column chromatography (SiO_2_, cyclohexane/Et_2_O 98:2) afforded **54a** (1.492 g, 6.97 mmol, 82%) as a colorless liquid. ***R*_f_ **= 0.55 (cyclohexane/Et_2_O 90:10); **^1^H NMR** (400 MHz, CDCl_3_) δ 7.58 (dd, *J* = 7.8, 1.1 Hz, 1H; Ar─H), 7.34–7.42 (m, 1H; Ar─H), 7.26–7.32 (m, 1H; Ar─H), 7.21–7.26 (m, 1H; Ar─H), 4.33 (q, *J* = 7.2 Hz, 2H; CH_2_), 2.43 (appt, *J *= 2.1 Hz, 3H; CH_3_), 1.31 (t, *J* = 7.2 Hz, 3H; CH_3_) ppm; **^19^F NMR** (470 MHz, CDCl_3_) δ −101.3 (s, 2F) ppm; **^19^F{^1^H} NMR** (470 MHz) δ −101.3 (s, 2F) ppm; **^13^C{^1^H} NMR** (101 MHz, CDCl_3_) δ 164.1 (t, *J* = 36.3 Hz; CO), 136.4 (t, *J* = 2.9 Hz, C_Ar_; C_2_), 131.8 (CH_Ar_; C_4_), 131.1 (t, *J* = 23.3 Hz, C_Ar_; C_1_), 130.7 (CH_Ar_; C_3_), 126.1 (t, *J* = 8.7 Hz, CH_Ar_; C_6_), 125.9 (CH_Ar_; C_5_), 114.2 (t, *J* = 251.8 Hz; CF_2_), 63.0 (CH_2_), 19.5 (t, *J* = 2.5 Hz; ArCH_3_), 13.8 (CH_3_) ppm; **IR** (neat): 2987 (w), 1762 (s), 1460 (m), 1284 (s), 1251 (s), 1093 (s), 1015 (s), 742 (s) cm^−1^; **LRMS** (EI, 70 eV) *m/z* (%): 214 (9) [*M*]^+^, 141 (100) [*M*‐COOEt]^+^, 91 (12) [C_7_H_7_]^+^; **HRMS** (ESI+): compound decomposed on spectrometer.

#### 4.2.22 Synthesis of Methyl 3‐((2‐methylbicyclo[1.1.1]pentan‐1‐yl)sulfonyl)Propanoate **55a**

To a solution of **35a** (73.8 mg, 0.37 mmol, 1 equiv.) in DCM (5 mL) was added portion‐wise *m‐*CPBA (179.4 mg, 1.0 mmol, 2.8 equiv.) at 0 °C. After complete addition, the reaction mixture was stirred 1 hour at r.t. and then poured into a saturated Na_2_SO_3_ solution (6 mL). The precipitate was filtered off and washed with additional DCM (2 × 6 mL). The phases were separated, the organic phase was washed with 1 M NaOH solution (10 mL), dried with Na_2_SO_4_, filtered, and concentrated. Purification through flash chromatography (*n*‐pentane/Et_2_O 65:35) afforded **55a** (68.5 mg, 0.29 mmol, 80%) as a colorless liquid. ***R*_f_** = 0.3 (*n*‐pentane:Et_2_O 65:35); **^1^H NMR** (400 MHz, CDCl_3_) δ 3.74 (s, 3H; OCH_3_), 3.16–3.21 (m, 2H; CH_2_), 2.83–2.88 (m, 2H; CH_2_), 2.81 (dd, *J* = 9.6, 3.0 Hz, 1H; H^B^), 2.75 (appquin, *J *= 6.3 Hz, 1H; H^A^), 2.61 (s, 1H; CH), 2.25 (dd, *J* = 6.2, 2.9 Hz, 1H; H^C^), 2.25 (d, *J* = 1.7 Hz, 1H; H^D^), 2.03 (dd, *J* = 9.7, 2.0 Hz, 1H; H^E^), 1.35 (d, *J* = 6.4 Hz, 3H; CH_3_) ppm;**^13^C{^1^H} NMR** (101 MHz, CDCl_3_) δ 171.0 (CO), 58.1 (C_2_), 55.8 (C), 52.4 (OCH_3_), 50.3 (C_5_), 46.1 (C_4_), 45.6 (CH_2_), 30.7 (CH), 25.8 (CH_2_), 10.6 (CH_3_) ppm; **IR** (neat): 2980 (w), 1736 (s), 1301 (s), 1207 (s), 1153 (s), 1125 (s) cm^−1^; **HRMS** (ESI+): *m/z* calcd for C_10_H_16_NaO_4_S 255.0662 [*M *+ Na]^+^; found 255.0656 (2.1 ppm error).

#### 4.2.23 Synthesis of Sodium 2‐methylbicyclo[1.1.1]Pentane‐1‐sulfinate **56a**

To a flask containing **55a** (55 mg, 0.24 mmol, 1.0 equiv.) was added THF (1 mL). To this solution, NaOMe (25 wt% in MeOH, 0.05 mL) was added at room temperature. The reaction was stirred for 30 minutes, and then the solvent was evaporated to give an inseparable mixture of **56a** (32 mg, 0.19 mmol, 81% (NMR)) and 3‐methoxypropionic acid in a ratio of 64:36 (^1^H NMR analysis) as a pale white solid. **^1^H NMR** (400 MHz, D_2_O) δ 2.44 (s, 1H; CH), 2.43 (dd, *J* = 9.7, 2.7 Hz, 1H; H^B^), 2.35 (appquin, *J* = 6.4 Hz, 1H; H^A^), 1.79 (d, *J* = 1.3 Hz, 1H; H^D^), 1.79 (dd, *J* = 6.1, 2.8 Hz, 1H; H^C^), 1.60 (dd, *J* = 9.5, 1.6 Hz, 1H; H^E^), 1.12 (d, *J* = 6.4 Hz, 3H; CH_3_) ppm; **^13^C{^1^H} NMR** (101 MHz, acetone‐*d*_6_) δ 61.6 (CSO_2_), 56.6 (C_2_), 47.6 (C_5_), 43.7 (C_4_), 31.1 (CH), 11.2 (CH_3_) ppm; **IR** (neat): 3287 (br), 2948 (w), 1562 (s), 1418 (s); **HRMS** (ESI‐): *m/z* calcd for C_6_H_9_O_2_S 145.0329 [*M*‐Na]^+^; found 145.0330 (0.7 ppm error).

## Conflict of Interest

The authors declare no conflict of interest.

## Supporting information



Supporting Information

Supporting Information

## Data Availability

The data that support the findings of this study are available in the supplementary material of this article.
