# Supplementary material for: Methyl on the Bridge: 2‐Methyl Propellane as Precursor for 1,3‐Substituted 2‐Methylated Bicyclo[1.1.1]Pentanes
Source: Chemistry. 2025 Oct 15;32(28):e02396. doi: 10.1002/chem.202502396 (PMC13411385; doi:10.1002/chem.202502396)
Supplement: Supplementary file 1 — Supporting Information [file CHEM-32-e02396-s001.pdf]

SUPPORTING INFORMATION

Methyl on the Bridge: 2-Methyl[1.1.1]propellane as Precursor for Substituted 2-Methylated Bicyclo[1.1.1]pentanes

Sean Verschaeve, Mariana Manso, Tibo Van Eeckhoorn, Mark Light, and Bruno Linclau

Table of contents

1 Experimental log*P* data 6
1.1 Determination of log*P* 6
1.2 Standard NMR settings 6
1.3 NMR processing 7
1.4 T1 measurements 7
1.5 LogP references 7
1.6 Data of all individual log*P* measurements 9
1.6.1 log*P* determination of **46a** 9
1.6.2 LogP determination of **46b** 9
1.6.3 log*P* determination of **47a** 10
1.6.4 log*P* determination of **47b** 10
1.7 clogP calculations 11
1.8 Example of NMR spectra for log*P* determination 12
1.8.1 Octanol sample **47b** before processing (**Figure S1**) 12
1.8.2 Octanol sample **47b** after processing (**Figure S2**) 13
1.8.3 Water sample **47b** before processing (**Figure S3**) 14
1.8.4 Water sample **47b** after processing (**Figure S4**) 15
2 NMR characterisation of 2-methyl[1.1.1]propellane 23a 16
2.1.1 ^{1}H NMR – Scalar coupling constants analysis 16
2.1.2 ^{13}C{^{1}H} NMR 17
3 Synthesis 19
3.1 General Methods. 19
3.2 Compounds available 19
3.3 Unsuccessful syntheses of novel compounds 20

3.3.1 Unsuccessful selective synthesis of 1-chloro-2-(chloromethyl)but-2-ene **25** 20
3.3.2 Unsuccessful Appel deoxychlorination of **28** 20
3.3.3 Unsuccessful Vilsmeier-Haack deoxychlorination of **28** 21
3.4 Unoptimised and alternative reactions – Synthesis of novel compounds 22
3.4.1 Synthesis of 1,1-dibromo-2,2-bis(chloromethyl)-3-methylcyclopropane **22** 22
3.4.2 Synthesis of (2,2-dibromo-3-methylcyclopropane-1,1-diyl)dimethanol **28** 23
3.4.3 Synthesis (2-bromo-3-methylcyclopropane-1,1-diyl)dimethanol **29** 24
3.4.4 Synthesis of (2-methylcyclopropane-1,1-diyl)dimethanol **30** 25
3.4.5 Synthesis of 2-(prop-1-en-1-ylidene)propane-1,3,-diol **31** 26
3.4.6 Synthesis of 1,1-dibromo-2-methyl-5,7-dioxa6-thiaspiro[2.5]octane-6-oxide **32** 26
3.4.7 Synthesis of 1,1-dibromo-2-methyl-5,7-dioxaspiro[2.5]octan-6-one **33** as byproduct of **22** .. 27
3.5 Synthesis of reported compounds 28
3.5.1 Synthesis of [1.1.1]propellane **23b** 28
3.5.2 Synthesis of bicyclo[1.1.1]pentan-1-yl(phenyl)sulfane **34b** 29
3.5.3 Synthesis of methyl 3-(bicyclo[1.1.1]pentan-1-ylthio)propanoate **35b** 29
3.5.4 Synthesis of *N*,*N*-dibenzylbicyclo[1.1.1]pentan-1-amine **36b** 30
3.5.5 Synthesis of 1,1'-(bicyclo[1.1.1]pentane-1,3-diyl)bis(ethan-1-one) **37b** 31
3.5.6 Synthesis of ethyl 2,2-difluoro-2-(3-iodobicyclo[1.1.1]pentan-1-yl)acetate **38b** 32
3.5.7 Synthesis of 2-(3-iodobicyclo[1.1.1]pentan-1-yl)acetamide **39b** 32
3.5.8 Synthesis of 2-(bicyclo[1.1.1]pentan-1-yl)acetamide **43b** 33
3.5.9 Synthesis of 1-((4-nitrophenyl)sulfonyl)bicyclo[1.1.1]pentane **49b** 33
3.5.10 Synthesis of methyl 3-(bicyclo[1.1.1]pentan-1-ylsulfonyl)propanoate **55b** 34
3.5.11 Synthesis of sodium bicyclo[1.1.1]pentane-1-sulfinate **56b** 35
4 NMR spectra reported compounds synthesised by a new method 36
4.1 1-Chloro-2-(chloromethyl)but-2-ene 25 36
4.1.1 1-Chloro-2-(chloromethyl)but-2-ene **25** (^{1}H NMR, $CDCl_3$, 400 MHz) 36
4.1.2 1-Chloro-2-(chloromethyl)but-2-ene **25** ($^{13}C\{^{1}H\}$ NMR APT, $CDCl_3$, 101 MHz) 37
5 NMR spectra for novel compounds 38
5.1 1,1-Dibromo-2,2-bis(chloromethyl)-3-methylcyclopropane 22 38
5.1.1 1,1-Dibromo-2,2-bis(chloromethyl)-3-methylcyclopropane **22** (^{1}H NMR, C_6D_6, 400 MHz) 38
5.1.2 1,1-Dibromo-2,2-bis(chloromethyl)-3-methylcyclopropane **22** ($^{13}C\{^{1}H\}$ NMR APT, C_6D_6, 101 MHz) 39
5.2 2-Methyl[1.1.1]propellane 23a 40
5.2.1 2-Methyl[1.1.1]propellane **23a** (^{1}H NMR, $CDCl_3$, 400 MHz) 40
5.2.2 2-Methyl[1.1.1]propellane **23a** ($^{13}C\{^{1}H\}$ NMR, $CDCl_3$, 101 MHz) 41
5.3 (2,2-Dibromo-3-methylcyclopropane-1-diyl)dimethanol 28 42
5.3.1 (2,2-Dibromo-3-methylcyclopropane-1-diyl)dimethanol **28** (^{1}H NMR, methanol-d_4, 400 MHz) 42

5.3.2 (2,2-Dibromo-3-methylcyclopropane-1-diyl)dimethanol (**29**) ($^{13}C\{^1H\}$ NMR APT, methanol-d_4, 101 MHz) 43

5.4 (2-Bromo-3-methylcyclopropane-1-diyl)dimethanol 29 44

5.4.1 (2-Bromo-3-methylcyclopropane-1-diyl)dimethanol **29** (^1H NMR, methanol-d_4, 500 MHz) 44

5.4.2 (2-Bromo-3-methylcyclopropane-1-diyl)dimethanol **29** ($^{13}C\{^1H\}$ NMR APT, methanol-d_4, 101 MHz) 45

5.5 (2-Methylcyclopropane-1,1-diyl)dimethanol 30 & 2-(prop-1-en-1-ylidene)propane-1,3-diol 31 46

5.5.1 (2-Methylcyclopropane-1,1-diyl)dimethanol **30** & 2-(prop-1-en-1-ylidene)propane-1,3-diol **31** (^1H NMR, methanol-d_4, 500 MHz) 46

5.5.2 (2-Methylcyclopropane-1,1-diyl)dimethanol **30** & 2-(prop-1-en-1-ylidene)propane-1,3-diol **3**1 ($^{13}C\{^1H\}$ NMR APT, methanol-d_4, 101 MHz) 47

5.6 1,1-Dibromo-2-methyl-5,7-dioxa-6-thiaspiro[2.5]octane-6-oxide 32 48

5.6.1 1,1-Dibromo-2-methyl-5,7-dioxa-6-thiaspiro[2.5]octane-6-oxide **32** (^1H NMR, $CDCl_3$, 400 MHz) 48

5.6.2 1,1-Dibromo-2-methyl-5,7-dioxa-6-thiaspiro[2.5]octane-6-oxide **32** ($^{13}C\{^1H\}$ NMR APT, $CDCl_3$, 101 MHz) 49

5.7 1,1-Dibromo-2-methyl-5,7-dioxaspiro[2.5]octan-6-one 33 50

5.7.1 1,1-Dibromo-2-methyl-5,7-dioxaspiro[2.5]octan-6-one **33** (^1H NMR, $CDCl_3$, 500 MHz) 50

5.7.2 1,1-Dibromo-2-methyl-5,7-dioxaspiro[2.5]octan-6-one **33** ($^{13}C\{^1H\}$ NMR APT, $CDCl_3$, 101 MHz) 51

5.8 (2-Methylbicyclo[1.1.1]pentan-1-yl)(phenyl)sulfane 34a 52

5.8.1 (2-Methylbicyclo[1.1.1]pentan-1-yl)(phenyl)sulfane **34a** (^1H NMR, $CDCl_3$, 400 MHz) 52

5.8.2 (2-Methylbicyclo[1.1.1]pentan-1-yl)(phenyl)sulfane **34a** ($^{13}C\{^1H\}$ NMR, $CDCl_3$, 101 MHz) 53

5.9 Methyl 3-(2-methylbicyclo[1.1.1]pentan-1-yl)thio)propanoate 35a 54

5.9.1 Methyl 3-(2-methylbicyclo[1.1.1]pentan-1-yl)thio)propanoate **35a** (^1H NMR, $CDCl_3$, 400 MHz) 54

5.9.2 Methyl 3-(2-methylbicyclo[1.1.1]pentan-1-yl)thio)propanoate **35a** ($^{13}C\{^1H\}$ NMR APT, $CDCl_3$, 101 MHz) 55

5.10 *N,N*-dibenzyl-2-methylbicyclo[1.1.1]pentan-1-amine 36a 56

5.10.1 *N,N*-dibenzyl-2-methylbicyclo[1.1.1]pentan-1-amine **36a** (^1H NMR, $CDCl_3$, 400 MHz) 56

5.10.2 *N,N*-dibenzyl-2-methylbicyclo[1.1.1]pentan-1-amine **36a** ($^{13}C\{^1H\}$ NMR, $CDCl_3$, 101 MHz) 57

5.11 1,1'-(2-Methylbicyclo[1.1.1]pentane-1,3-diyl)bis(ethan-1-one) 37a 58

5.11.1 1,1'-(2-Methylbicyclo[1.1.1]pentane-1,3-diyl)bis(ethan-1-one) **37a** (^1H NMR, $CDCl_3$, 400 MHz) 58

5.11.2 1,1'-(2-Methylbicyclo[1.1.1]pentane-1,3-diyl)bis(ethan-1-one) **37a** ($^{13}C\{^1H\}$ NMR APT, $CDCl_3$, 101 MHz) 59

5.12 Ethyl 2,2-difluoro-2-(3-iodo-2-methylbicyclo[1.1.1]pentan-1-yl)acetate 38a 60

5.12.1 Ethyl 2,2-difluoro-2-(3-iodo-2-methylbicyclo[1.1.1]pentan-1-yl)acetate **38a** (^1H NMR, $CDCl_3$, 400 MHz) 60

5.12.2 Ethyl 2,2-difluoro-2-(3-iodo-2-methylbicyclo[1.1.1]pentan-1-yl) **38a** ($^{19}F\{^{1}H\}$ NMR, $CDCl_3$, 376 MHz) 61

5.12.3 Ethyl 2,2-difluoro-2-(3-iodo-2-methylbicyclo[1.1.1]pentan-1-yl)acetate **38a** ($^{13}C\{^{1}H\}$ NMR, $CDCl_3$, 101 MHz) 62

5.13 2-(3-Iodo-2-methylbicyclo[1.1.1]pentan-1-yl)acetamide 39a 63

5.13.1 2-(3-Iodo-2-methylbicyclo[1.1.1]pentan-1-yl)acetamide **39a** (crude ^{1}H NMR spectrum, $CDCl_3$, 400 MHz) 63

5.14 2-(3-Ethylidene-1-iodocyclobutyl)acetamide 40 64

5.14.1 2-(3-Ethylidene-1-iodocyclobutyl)acetamide **40** (^{1}H NMR, $CDCl_3$, 400 MHz) 64

5.14.2 2-(3-Ethylidene-1-iodocyclobutyl)acetamide **40** ($^{13}C\{^{1}H\}$ NMR, $CDCl_3$, 101 MHz) 65

5.15 2-(2-Methylbicyclo[1.1.1]pentan-1-yl)acetamide 43a 66

5.15.1 2-(2-Methylbicyclo[1.1.1]pentan-1-yl)acetamide **43a** ^{1}H NMR, $CDCl_3$, 500 MHz) 66

5.15.2 2-(2-Methylbicyclo[1.1.1]pentan-1-yl)acetamide **43a** ($^{13}C\{^{1}H\}$ NMR APT, $CDCl_3$, 101 MHz) 67

5.16 Methyl *N*-(*tert*-butoxycarbonyl)-2-methylbicyclo[1.1.1]pentan-1-yl)-L-cysteinate 44 68

5.16.1 Methyl *N*-(*tert*-butoxycarbonyl)-2-methylbicyclo[1.1.1]pentan-1-yl)-L-cysteinate **44** (^{1}H NMR, $CDCl_3$, 400 MHz) 68

5.16.2 Methyl *N*-(*tert*-butoxycarbonyl)-2-methylbicyclo[1.1.1]pentan-1-yl)-L-cysteinate **44** ($^{13}C\{^{1}H\}$ NMR, $CDCl_3$, 101 MHz) 69

5.17 (2-Methylbicyclo[1.1.1]pentanyl)(phenyl)sulfoximine 45 70

5.17.1 (2-Methylbicyclo[1.1.1]pentanyl)(phenyl)sulfoximine **45** (^{1}H NMR, $CDCl_3$, 400 MHz) 70

5.17.2 (2-methylbicyclo[1.1.1]pentanyl)(phenyl)sulfoximine **45** ($^{13}C\{^{1}H\}$ NMR, $CDCl_3$, 101 MHz) 71

5.18 2,2-Difluoro-2-(2-methylbicyclo[1.1.1]pentan-1-yl)ethan-1-ol 46a 72

5.18.1 2,2-Difluoro-2-(2-methylbicyclo[1.1.1]pentan-1-yl)ethan-1-ol **46a** (^{1}H NMR, DCM-d_2, 400 MHz) 72

5.18.2 2,2-Difluoro-2-(2-methylbicyclo[1.1.1]pentan-1-yl)ethan-1-ol **46a** ($^{19}F\{^{1}H\}$ NMR, DCM-d_2, 376 MHz) 73

5.18.3 2,2-Difluoro-2-(2-methylbicyclo[1.1.1]pentan-1-yl)ethan-1-ol **46a** (^{19}F NMR, DCM-d_2, 376 MHz) 74

5.18.4 2,2-Difluoro-2-(2-methylbicyclo[1.1.1]pentan-1-yl)ethan-1-ol **46a** ($^{13}C\{^{1}H\}$ NMR, DCM-d_2, 101 MHz) 75

5.19 2-(Bicyclo[1.1.1]pentan-1-yl)-2,2-difluoroethan-1-ol 46b 76

5.19.1 2-(Bicyclo[1.1.1]pentan-1-yl)-2,2-difluoroethanol-1-ol **46b** (^{1}H NMR, $CDCl_3$, 500 MHz) 76

5.19.2 2-(Bicyclo[1.1.1]pentan-1-yl)-2,2-difluoroethanol-1-ol **46b** ($^{19}F\{^{1}H\}$ NMR, $CDCl_3$, 470 MHz)... 77

5.19.3 2-(Bicyclo[1.1.1]pentan-1-yl)-2,2-difluoroethanol-1-ol **46b** (^{19}F NMR, $CDCl_3$, 470 MHz) 78

5.19.4 2-(Bicyclo[1.1.1]pentan-1-yl)-2,2-difluoroethanol-1-ol **46b** ($^{13}C\{^{1}H\}$ NMR APT, $CDCl_3$, 101 MHz) 79

5.20 2,2-Difluoro-2-(*o*-toly)ethan-1-ol 47a 80

5.20.1 2,2-Difluoro-2-(*o*-tolyl)ethan-1-ol **47a** (^{1}H NMR, $CDCl_3$, 400 MHz) 80

5.20.2 2,2-Difluoro-2-(*o*-tolyl)ethan-1-ol **47a** ($^{19}F\{^{1}H\}$ NMR, $CDCl_3$, 470 MHz) 81

5.20.3 2,2-Difluoro-2-(*o*-tolyl)ethan-1-ol **47a** (^{19}F NMR, $CDCl_3$, 470 MHz) 82

5.20.4 2,2-Difluoro-2-(*o*-tolyl)ethan-1-ol **47a** ($^{13}C\{^{1}H\}$ NMR, $CDCl_3$, 101 MHz) 83
5.21 2,2-Difluoro-2-phenylethan-1-ol 47b 84
5.21.1 2,2-Difluoro-2-phenylethan-1-ol **47b** (^{1}H NMR, $CDCl_3$, 400 MHz) 84
5.21.2 2,2-Difluoro-2-phenylethan-1-ol **47b** (^{19}F NMR, $CDCl_3$, 470 MHz) 85
5.21.3 2,2-Difluoro-2-phenylethan-1-ol **47b** ($^{19}F\{^{1}H\}$ NMR, $CDCl_3$, 470 MHz) 86
5.21.4 2,2-Difluoro-2-phenylethan-1-ol **47b** ($^{13}C\{^{1}H\}$ NMR APT, $CDCl_3$, 101 MHz) 87
5.22 2-Methyl-1-((4-nitrophenyl)sulfonyl)bicyclo[1.1.1]pentane 49a 88
5.22.1 2-Methyl-1-((4-nitrophenyl)sulfonyl)bicyclo[1.1.1]pentane **49a** (^{1}H NMR, $CDCl_3$, 400 MHz) .. 88
5.22.2 2-Methyl-1-((4-nitrophenyl)sulfonyl)bicyclo[1.1.1]pentane **49a** ($^{13}C\{^{1}H\}$ NMR APT, $CDCl_3$, 101 MHz) 89
5.23 Ethyl 2,2-difluoro-2-(*o*-tolyl)acetate 54a 90
5.23.1 Ethyl 2,2-difluoro-2-(*o*-tolyl)acetate **54a** (^{1}H NMR, $CDCl_3$, 400 MHz) 90
5.23.2 Ethyl 2,2-difluoro-2-(*o*-tolyl)acetate **54a** ($^{19}F\{^{1}H\}$ NMR, $CDCl_3$, 470 MHz) 91
5.23.3 Ethyl 2,2-difluoro-2-(*o*-tolyl)acetate **54a** (^{19}F NMR, $CDCl_3$, 470 MHz) 92
5.23.4 Ethyl 2,2-difluoro-2-(*o*-tolyl)acetate **54a** ($^{13}C\{^{1}H\}$ NMR APT, $CDCl_3$, 101 MHz) 93
5.24 Methyl 3-((2-methylbicyclo[1.1.1]pentan-1-yl)sulfonyl)propanoate 55a 94
5.24.1 Methyl 3-((2-methylbicyclo[1.1.1]pentan-1-yl)sulfonyl)propanoate **55a** (^{1}H NMR, $CDCl_3$, 400 MHz) 94
5.24.2 Methyl 3-((2-methylbicyclo[1.1.1]pentan-1-yl)sulfonyl)propanoate **55a** ($^{13}C\{^{1}H\}$ NMR, $CDCl_3$, 101 MHz) 95
5.25 Sodium 2-methylbicyclo[1.1.1]pentane-1-sulfinate 56a 96
5.25.1 Sodium 2-methylbicyclo[1.1.1]pentane-1-sulfinate **56a** (^{1}H NMR, D_2O, 400 MHz) 96
5.25.2 Sodium 2-methylbicyclo[1.1.1]pentane-1-sulfinate **56a** ($^{13}C\{^{1}H\}$ NMR, acetone-d_6, 101 MHz) 97
6 X-ray data 98
6.1 Crystal data for compound 49a 98
6.1.1 Crystal data for compound **49b** 104
7 References 108

1 Experimental log*P* data

1.1 Determination of log*P*

Lipophilicities were determined using a previously published protocol, which was slightly adapted.[1] The compound of interest (= COI) is weighed (15-25 mg) in a vial (= vial 1). The reference, with known log*P* close to the clog*P* of the compound, is weighed in a second vial (= vial 2). Beware of volatility. Seal the vial(s) between steps if necessary to minimise loss.

If the COI is more soluble in octanol, 6.5 ml of octanol is added to vial 1 and stirred between 10-15 minutes to ensure complete dissolution. Vial 1 is added over vial 2 and this is stirred for 15 min. Three oven-dried pear-shaped flasks, containing an oven-dried stirrer bar, were sealed with a septum and allowed to cool down to 25 °C. To each flask, 2 ml of the mixture of COI and reference in octanol is transferred. Similarly, 2 ml of water is transferred to each flask. The resulting three, biphasic mixtures are stirred (at 600 rpm) for 2 h at 25 °C, and then left without stirring for at least 16 h at 25 °C to allow phase separation.

If the COI is more soluble in water, 6.5 ml of water is added to vial 1 and stirred between 10-15 minutes to ensure complete dissolution. Vial 1 is added over vial 2 and this is stirred for 15 min.

Three oven-dried pear-shaped flasks, containing an oven dried stirrer bar, were sealed with a septum and allowed to cool down to 25 °C. To each flask, 2 ml of octanol is transferred. Similarly, 2 ml of the mixture of COI and reference in water is transferred to each flask. The resulting three, biphasic mixtures are stirred (at 600 rpm) for 2 h at 25 °C, and then left without stirring for at least 16 h at 25 °C to allow phase separation.

NMR sample preparation: For each flask, an aliquot of 0.5 mL was taken from each phase using 1 mL syringes with long needles and added to two separate screw-cap NMR tubes (6 tubes in total: 3 × water and 3 x octanol). 0.1 ml of acetone-d_6 was added to each NMR tube and mixed by inversion (20-30 times). The NMR tubes were sealed with the screw-cap to prevent evaporation of the solvent and compounds.

As such, a statistically relevant number of measurements are performed: three per phase. Any changes to this general protocol are mentioned in the respective sections below.

1.2 Standard NMR settings

For $^{19}F\{^1H\}$ NMR experiments, first, 1 octanol and 1 water sample were analysed with the following NMR parameters as calibration of the signal-to-noise ratio (S/N ratio): SW = 200 ppm; centred frequency offset (o1p) between two diagnostic fluorine resonances; NS = 16; D1 = 30 s (octanol sample), D1 = 60 s (water sample). If needed, an increased number of transients (NS) for a good S/N ratio (typically >150) was applied. All six NMR tubes were now analysed with the adapted NMR parameters. After NMR data processing, integration ratios ρ_{oct} and ρ_{aq} were determined, where ρ_{oct} is defined as the integration ratio

between the compound and the reference compound in the octanol sample, likewise for ρ_{aq}. These were used in the equation ($\log(P_{COI}) = \log(P_{ref}) + \log(\rho_{oct}/\rho_{aq})$) to obtain the log*P* value. The mean of the three log*P* values is then defined as the mean log*P* value of the compound.

1.3 NMR processing

FIDs were processed using ACD/Labs NMR software. The obtained FID file was reprocessed using the following conditions: Wfunction (LB 2 Exponential), Zero Filling (the initial value was multiplied by 4, *e.g.,* 65536 to 262144), and then Fourier transformed, followed by mouse phasing. The baseline was corrected by spectrum averaging, Set Points, with a Box Half Width = 500, Auto Create Curve, and Cubic Spline. The integration ratio was obtained by manual integration. Bias correction could be applied by adjusting the tilt and slope if the integration curve is not parallel to the baseline. A general integration window was set at 4.0 ppm (± 2.0 ppm from resonance). In cases where impurities were found in the integration window, the latter was adjusted to exclude the impurities.

1.4 T1 measurements

A small amount of the COI or the reference of choice was added to an NMR tube. 0.5 mL of *n*-octanol or water was added to the tube, followed by 0.1 mL of acetone-d_6 for deuterium locking. The tube was inverted 20 times to homogenise the mixture. Optionally, the tube was sonicated for 5 minutes. The tube was inserted in the magnet, followed by automated locking, ATM, and gradient shimming. An inversion-recovery experiment for ^{19}F nuclei was performed (SW = 20 ppm, o1p centred to the chemical shift of the resonance of interest).

The FID was processed using TopSpin (manual phasing, automated baseline correction, manual peak picking) and analysed using the Bruker Dynamics Center. The T1 was calculated by Monte Carlo simulation with a 95% confidence interval.

1.5 LogP references

A series of references with known log*P* were used. Below, Table S1 summarises experimental data relevant for the log*P* experiments.

Table S1. Summary of the log*P* references with their respective data.

**Reference**	δ_{oct} (ppm)	δ_{water} (ppm)	$T1_{oct}$ (s)	$T1_{water}$ (s)	log*P*
2-fluorophenol	- 137.7	- 137.8	1.90	3.46	+1.70[1]

4,4,4-trifluorobutan-1-ol	- 66.9	- 66.0	1.67	2.62	+0.91[1]
Hexafluoro-*i*-propanol	- 76.2	- 75.7	1.69	2.89	+1.69[1]

1.6 Data of all individual log*P* measurements

1.6.1 log*P* determination of **46a**

2-Fluorophenol was used as reference.

Compound	Experiment	D1 (s)	S/N_{Ref}	S/N_{COI}[a]	ρ_{oct}/ρ_{water}	log*P*
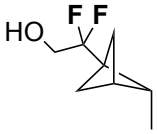	Oct 1	30	3249	3399	112.73	+2.123
	Water 1[b]	60	530	104[c]	42.55	
	Oct 2	30	3174	3399	112.79	+2.078
	Water 2[b]	60	433	89[c]	47.21	
	Oct 3	30	3222	3388	112.69	+2.134
	Water 3[b]	60	410	85[c]	41.50	
Average log*P*: +2.11		STDev: 0.029			Error: ±0.017	

[a] S/N_{COI} < 150. Due to NMR-measurement time restrictions.

[b] Change from standard in NMR parameter settings: NS = 2048.

[c] In *n*-octanol, only 1 resonance was observed for the 2 diastereotopic fluorines of **46a**. In water, 2 resonances (strongly roofed doublets) were observed, hence the lower S/N_{COI}.

1.6.2 LogP determination of **46b**

4,4,4-Trifluorobutan-1-ol was used as reference.

Compound	Experiment	D1 (s)	S/N_{Ref}	S/N_{COI}	ρ_{oct}/ρ_{water}	log*P*
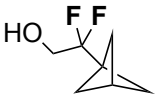	Oct 1	30	1887	1324	75.76	+1.562
	Water 1[a]	60	2555	389	16.89	
	Oct 2	30	1594	1235	75.96	+1.569
	Water 2[a]	60	2428	361	16.67	
	Oct 3	30	1914	1304	74.85	+1.571
	Water 3[a]	60	2512	380	16.35	
Average log*P*: +1.57		STDev: 0.005			Error: ±0.003	

[a] Change from standard in NMR parameter settings: NS = 1024.

1.6.3 log*P* determination of **47a**

Hexafluoro-*i*-propanol was used as reference.

Compound	Experiment	D1 (s)	S/N_{Ref}	S/N_{COI}	ρ_{oct}/ρ_{water}	log*P*
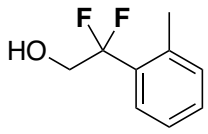	Oct 1	30	3889	2359	65.63	+2.036
	Water 1[a]	15[b]	897	257	29.56	
	Oct 2	30	4008	2416	65.81	+2.014
	Water 2[a]	15[b]	842	238	31.20	
	Oct 3	30	3818	2317	65.48	+2.035
	Water 3[a]	15[b]	715	204	29.58	
Average log*P*: +2.03		STDev: 0.012		Error: ±0.007		

[a] Change from standard in NMR parameter settings: NS = 1024.

[b] Change from standard in NMR parameter settings: $D1_{water}$ = 15 s ($T1_{COI,\ water}$ = 1.05 s).

1.6.4 log*P* determination of **47b**

2-flurophenol was used as reference.

Compound	Experiment	D1 (s)	S/N_{Ref}	S/N_{COI}	ρ_{oct}/ρ_{water}	log*P*
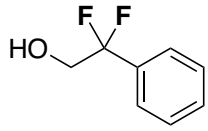	Oct 1	30	1347	1552	125.14	+1.582
	Water 1[a]	60	205	316	164.29	
	Oct 2	30	1140	1437	126.44	+1.571
	Water 2[a]	60	196	308	170.01	
	Oct 3	30	1194	1421	124.24	+1.567
	Water 3[a]	60	186	293	168.73	
Average log*P*: +1.57		STDev: 0.008		Error: ±0.004		

[a] Change from standard in NMR parameter settings: NS = 512.

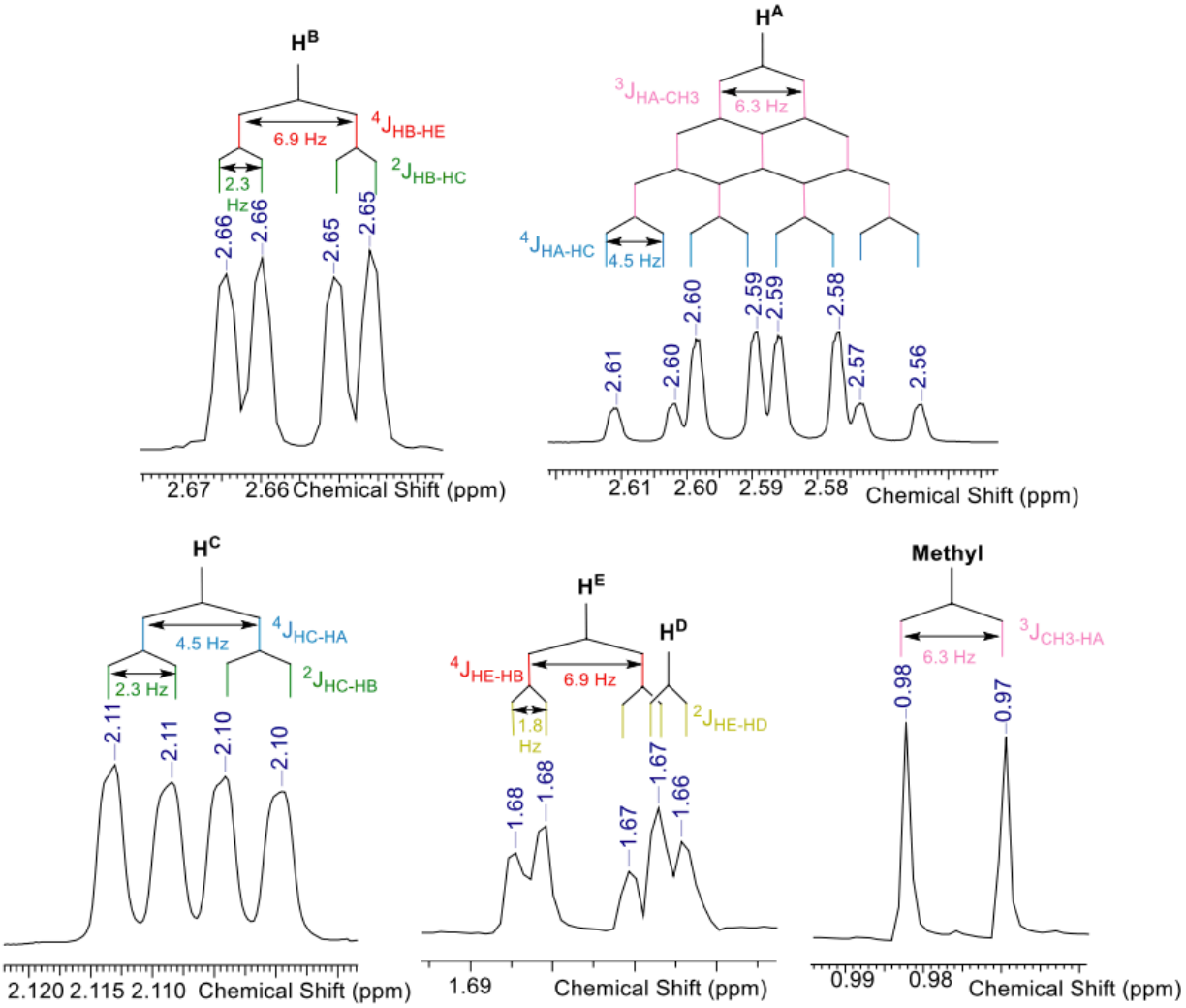

Figure S7. Zoom areas of the characteristic resonances of **23a**.

Table S4. Summary of scalar couplings in **23a**.

δ (ppm)	Protons	multiplicity	$^{X}J_{HY-HZ}$
2.66	H^B	dd	$^{4}J_{HB-HE}$ = 6.9 Hz; $^{2}J_{HB-HC}$ = 2.3 Hz
2.59	H^A	qd	$^{3}J_{HA-Me}$ = 6.3 Hz; $^{4}J_{HA-HC}$ = 4.5 Hz
2.11	H^C	dd	$^{4}J_{HC-HA}$ = 4.5 Hz; $^{2}J_{HC-HB}$ = 2.3 Hz
1.69	H^E	dd	$^{4}J_{HE-HB}$ = 6.9 Hz; $^{2}J_{HE-HD}$ = 1.8Hz
1.68	H^D	d	$^{2}J_{HD-HE}$ = 1.8 Hz
0.98	Me	d	$^{3}J_{Me-HA}$ = 6.3 Hz

2.1.2 $^{13}C\{^{1}H\}$ NMR

All carbons could be identified. The methylene carbons (C_4 and C_5), as well as C_2 and the methyl carbon, were easily identified by HSQC-DEPT. The bridgehead carbons (C_1 and C_3), despite being

3 Synthesis

3.1 General Methods.

All chemical reagents were obtained from commercial sources and used without further purification. Anhydrous solvents were purchased from commercial sources. Anhydrous solvents were distilled immediately prior to use, except for anhydrous DMF which was purchased in sealed containers from commercial sources. Et_2O and THF was distilled from Na/benzophenone immediately prior to use. DCM and Et_3N were dried over CaH_2. All glassware was flame-dried under vacuum and cooled under N_2 prior to use. Water or air-sensitive reactions were performed under inert atmosphere, using dry solvents. Reactions were monitored by TLC (Merck Kieselgel 60 F254, aluminium sheet). Column chromatography was performed on silica gel (60 Å, particle size 35-70 μm). All reported solvent mixtures are volume measures. Reactions *in flow* were carried out using a peristatic pump (Vapourtec SF-10, blue pump tube) and a home-built flow reactor (spiral PTFE tubing 0.8mm ID x 1.6 mm OD, internal volume of 4 mL). Photochemical reactions were carried out using 6 UV-A LEDs assembled on a heatsink (Starboard, Lupinus UV-A, 365 nm, 130 ° beam angle, 5.25 W total power). Preparative RP-HPLC was carried out using a Phenomenex Luna Axia C_{18} column (250×22 mm, particle size 5 μm at 17.5 mL min^{-1}, 35 °C). ^{1}H, ^{19}F, ^{13}C NMR spectra were recorded at room temperature on a BRUKER AV400/500 spectrometer. ^{1}H and ^{13}C chemical shifts (δ) are quoted in ppm relative to residual solvent peaks as appropriate. ^{19}F spectra were externally referenced to $CFCl_3$. The coupling constants (*J*) are given in Hertz (Hz). The proton NMR signals were designated as follows: s (singlet), d (doublet), t (triplet), q (quartet), quin (quintet), sxt (sextet), spt (septet), m (multiplet), br (broad), app (apparent) or a combination of the above. IR spectra were recorded as neat films on a Nicolet 380 FT-IR and a Perkin-Elmer Spectrum 1000 FT-IR spectrometer. Absorption peaks are given in cm^{-1} and the intensities were designated as follows: w (weak), m (medium), s (strong), br (broad). Melting points were recorded on a Reichert melting point apparatus, equipped with a Reichert microscope. Low-resolution ES mass spectra were recorded on a WATERS ZMD single quadrupole system. High-resolution mass spectra were recorded on a Bruker Apex III FT-ICR-MS and Agilent 6220a series TOF (LC-HRMS) or Thermo MAT 900 XP double-focusing sector field (GC HRMS).

3.2 Compounds available

The log*P* references were commercially available and used without further purification.
